# The Assessment of Dual-Cycle Identity Models Among Secondary School Students: The Hungarian Adaptation of DIDS and U-MICS

**DOI:** 10.3389/fpsyt.2022.804529

**Published:** 2022-03-21

**Authors:** Adrienn Rivnyák, Evelyn Járdaházi, Nikolett Arató, Bernadette Péley, András Láng

**Affiliations:** Institute of Psychology, University of Pécs, Pécs, Hungary

**Keywords:** adolescence, identity development process, identity status, U-MICS, DIDS, cultural validation

## Abstract

The aim of the present study was to evaluate the factor structure and validity of the Hungarian versions of the Dimensions for Identity Development Scale (DIDS) and Utrecht-Management of Identity Commitments Scale (U-MICS). Both models assume that the iterative process of exploring and evolving commitments occurs in two distinct cycles. The sample for testing the factor structure of DIDS consisted of 808 adolescents (357 boys and 451 girls) aged between 14 and 21 years (M_age_ = 16.86; SD = 1.35). The sample for testing the factor structure of U-MICS consisted of 803 adolescents (353 boys and 450 girls) aged between 14 and 21 years (M_age_ = 16.88; SD = 1.34). Results indicated a five factor model of DIDS in the present sample. All the five dimensions correlated as hypothesized both internally and externally. In line with previous research, six clusters emerged based on the dimensions of DIDS, including ruminative moratorium. Regarding U-MICS, results indicated a three factor model in the present sample. All the three dimensions were internally and externally correlated as hypothesized regarding both ideological and interpersonal identity domains. With regard to the identity status cluster solution, five clusters emerged in both the educational and friendship domains. We found specific variation regarding identity clusters in the two identity domains. Our results support the use of these two measurements in Hungarian context. Further, our results confirm the divergent developmental dynamics of ideological and interpersonal identity domains.

## Introduction

Dual cycle models of identity development have strongly influenced the field of identity research on adolescence and emerging adulthood. These approaches provide a dynamic approach to identity processes. Both the five-dimensional model (1) and the three-dimensional model (2) of identity formation assume that the iterative process of exploring and evolving commitments occur in two distinct cycles. To our knowledge, no previous study has ever compared the processes and statuses based on these two approaches. Thus, the aims of the present study are two-fold. Primarily this paper discusses the methodological characteristics of the Hungarian adaptation of identity measurements based on the five- and three-dimensional model. Additionally, we make some theoretical proposals for consideration based on our result.

One of the most fundamental developmental tasks of adolescence is identity synthesis (3–5). Identity is the notion of who one is. It can be defined as the sense of personal uniqueness and the sense of self-sameness across different times and contexts (6). In his psychosocial theory, Erikson (7) assumed that identity formation was a lifelong process that already began in early childhood and became emphasized and conscious in adolescence, during the psychosocial crisis of identity vs. identity diffusion. At this stage of life, conflicts from earlier stages of development are revived, and previous identities and continuities become questioned. The childhood identifications are no longer sufficient, so the re-evaluation of them is necessary in order to be integrated at a more mature level. Furthermore, the increasingly wider social environment requires the individual to match individual and social identities. The result of these processes will form the sense of an integrated self (6, 7).

For decades, the most prominent model that operationalized Erikson's identity theory into measurable constructs was Marcia's (8, 9) identity status approach. Marcia introduced two qualitatively distinct dimensions of identity: crisis that was later called exploration and the concept of commitment. Exploration refers to “the adolescent's period of engagement in choosing among meaningful alternatives,” while commitment is defined as “the degree of personal investment the individual exhibits” [(8), p. 551]. Based on the presence or absence of these two main processes, Marcia (8) identified four distinct identity statuses: identity achievement (commitment after exploration), foreclosure (commitment without exploration), moratorium (exploring but not committed) and identity diffusion (no exploration and no commitment).

In the last decades, based on Marcia's (8) identity status approach, dual-cycle models of identity development emerged. The two most prominent models, that refined the dimension of exploration and commitment, are the five-dimensional (1) and the three-dimensional models (2). These models represent a more process-oriented approach, as they shift the focus on the formative and evaluation processes underlying identity statuses and their interrelated nature. Dual-cycle models have made a significant contribution to identity research. By identifying the underlying processes of commitment making and exploration, these dual-cycle approaches provide a more complex evaluation of developmental trajectories. Both theories consider identity formation as a dynamic and recurring process of exploration and making commitments, which occurs in two cycles. However, the two models assess identity formation is different domains. The model by Crocetti et al. (2) evaluates the formation of identity separately in specific areas such as education and friendship. In contrast, the model described by Luyckx et al. (1) captures identity development along future plans, which integrate specific context into a more general domain. Self-report measures are available for both models (1, 2). Different cultural adaptations supported the utility of both dual-cycle approaches (10–20).

Luyckx et al. (21) introduced a dynamic model of identity formation by dividing both exploration and commitment into two components. As a result of this, four interrelated identity processes were distinguished. These dimensions are commitment making, identification with commitment, broad exploration and deep exploration. The *commitmen*t dimension refers to whether the individual has already made a decision in identity relevant questions, while *identification with commitment* captures the process when adolescents identify with their choices and the commitment evolves into an integrated part of the self. *Exploration in breadth* refers to the mapping of different identity alternatives, which is an important facilitator of commitment making. By comparison, during *in depth explorations*, the individual collects information about current commitments, which makes it possible to assess the extent to which the choice meets the individual's own inner criteria (22). During the first cycle of identity formation, adolescent explore different alternatives and make some initial commitments. During the second cycle adolescents evaluate these initial commitments by exploring them in depth, and either identify with them or a new commitment formation cycle begins (21). Later, the model was extended with *ruminative exploration* as the fifth dimension that proved to be a significant risk factor regarding healthy identity development (1, 23). In contrast to reflective exploration processes, ruminative exploration is a maladaptive process characterized by continuous exploration without forming commitments (1). Ruminative moratorium was found to be associated with higher presence of depressive feelings and more negative and more unstable self-esteem (23). By unpacking exploration and commitment and introducing ruminative exploration, the five-dimensional model has the advantage of identifying more than four identity statuses.

Based on the five-dimensional model, Dimensions for Identity Development Scale (DIDS) was developed by Luyckx et al. (1). In line with the theoretical approach, DIDS assesses identity processes with five distinct scales in the domain of general future plans: commitment making, exploration in breadth, exploration in depth, identification with commitment, and ruminative exploration. In recent years, many cultural adaptations of DIDS were developed, for instance, German, Turkish, American, Swiss, Polish, Japanese, Greek, Georgian, and Finnish (13–20, 24). Although a number of the above mentioned studies confirmed the original five-dimensional model, six factor models emerged in French, Georgian and Finnish samples (13, 18, 19). Along similar lines in all three samples, the exploration in depth dimension proved to be inconsistent and had to be divided in two different dimensions. One dimension referred to the reflective nature of exploration in depth that strengthened commitments. This was consistent with the proposition of Luyckx et al. (1). In contrast, the other dimension referred to the questioning and revision of existing commitments. This idea was in line with Grotevant's (25) assumption that exploration can induce the questioning of commitments. Identity statuses can be empirically classified through cluster analysis based on the five dimensions. Luyckx et al. (1) identified altogether the following six clusters. The *achievement* status consisted of individuals who scored above average on both commitment dimensions, as well as on exploration in breadth and exploration in depth, and below average on ruminative exploration. Individuals with the *foreclosure* status scored above average on both commitments dimensions and below average on in breadth, in depth, and ruminative exploration. The moratorium status described by Marcia (8) did not emerge, but a ruminative moratorium status was identified. *Ruminative moratorium* composed of individuals who scored average on commitment dimensions, and above average on the exploration dimensions including ruminative exploration. Luyckx et al. (1) distinguished two diffusion clusters. Individuals with the *diffused diffusion* cluster had scores below average on both commitment dimensions, average scores on exploration in breadth and exploration in depth, and scores above average on ruminative moratorium. In comparison, individuals with the *carefree diffusion* status scored below average on both commitment dimensions, on in breadth and in depth exploration, and average on ruminative exploration. Although it seems that individuals with the carefree diffusion status ruminate less on future plans than their diffused diffused peers, both proved to be a risk group concerning psychosocial well-being (17). Finally, an undifferentiated cluster also emerged, in which individuals scored intermediate on all the five dimensions. Although fundamentally identical identity statuses emerged across nations and cultures, some differences in the distribution of identity statuses were revealed in empirical literature. In the study of Schwartz et al. (17) a searching moratorium cluster emerged. Searching moratorium was theoretically described by Meeus et al. (26); individuals in this status appeared to be exploring new alternatives while maintaining some of their prior commitments.

Crocetti et al. (2) proposed a three-factor model of identity development with emphasis on the formation, evaluation, and revision of identity elements in ideological and interpersonal identity domains. They assumed that adolescents already have preliminary commitments based on childhood identification when they enter adolescence (27). Consequently the first cycle is identity formation, during which adolescents evaluate their present commitments and compare them with potential alternatives. In case they feel their commitments to be no longer satisfactory, they start to revise them. The second cycle is identity maintenance, during which the focus shifts from finding new commitments to reflecting on and validating existing commitments (27). Three principal processes have been identified. *Commitment* refers to permanent and strong life choices, the gain of these choices will be self-confidence. *In depth exploration* refers to active exploration processes about the existing commitments by searching for further information and talking about them with significant others. Finally, *reconsideration of commitments* represents the comparison of existing commitments with new alternatives, when current commitments are no longer sufficient. High levels of reconsideration of commitments have been proved to be strongly related to depressive symptoms and to be negatively associated with self-concept clarity (2).

Utrecht-Management of Identity Commitments Scale (U-MICS) was developed to assess commitment, in depth exploration and reconsideration of commitments (2). An important advantage of this questionnaire is that it can be employed to assess identity formation processes distinctively in ideological (e.g., education) and relational (e.g., friendship) domains. Dynamics of identity formation can be different in different identity domains and can have different associations with outcomes of interest. It seems that global identity processes show low convergence of identity processes across distinct identity domains (28). Considering the dimensions of the instrument, numerous studies confirmed the three-factor structure of U-MICS in various countries including the Netherlands, Italy, Romania, Switzerland, Turkey, Poland, Bulgaria, Czech Republic, Kosovo, Slovenia, Portugal, China, Japan, Taiwan, Spain, and Israel (2, 10–12, 14, 19, 29–31).

Furthermore, identity statuses can be empirically classified through cluster analysis based on the three identity process. Crocetti et al. (32) distinguished five identity statuses on the sample of early and middle adolescent groups, four of which relied on the work of Marcia (8). Individuals with the *achievement* status typically had scores above average on commitment and in depth exploration and below average on reconsideration of commitment. Likewise, the *foreclosure* status was consisted of individuals with scores above average on commitment, but scored average on in depth exploration dimensions and below average on reconsideration. The *diffusion* status composed of individuals scored below average regarding commitment, in depth exploration and reconsideration of commitment. Individuals with the *moratorium* status scored below average on commitment, average on in depth exploration and above average on reconsideration of commitment. The fifth status has been titled *searching moratorium* and was separated from moratorium. Individuals with searching moratorium scored above average on both commitment and in depth exploration, just like on reconsideration of commitment. While moratorium considered representing the current struggle for finding satisfying commitment, the searching moratorium refers to the revision of existing commitments by looking for new alternatives (32).

The present study had four main objectives. First, we wanted to test to factor structure of the Hungarian versions of DIDS and U-MICS. With regard to DIDS, we tested six different models: four four-factor models, a five-factor model, and a six-factor model. The four-factor models and the five-factor model were based on the work by Luyckx et al. (1). The six-factor model was based on the validation study of the Finnish and Greek versions of DIDS (13, 20). We expected either the five- or the six-factor model to show the most adequate fit to our data. With regard to U-MICS, we tested three models based on Crocetti et al. (33) for both domain versions. We expected that the three-factor model would show adequate fit to our data for both domain versions.

Second, we wanted to reveal how H-DIDS and H-U-MICS would classify Hungarian adolescents. We expected that Hungarian adolescents would be classified into six and five clusters (for H-DIDS and H-U-MICS, respectively) that would be similar to those in previous studies [e.g., (1, 33–35)].

Third, we wanted to test the validity of H-DIDS and H-U-MICS. We did this on the level of variables and also in a person-centered approach. With regard to the variable-level approach, our expectations were based both on theoretical assumptions about the identity development process and on empirical results (for a summary see the corresponding sections of Introduction). We expected commitment to be positively associated with favorable psychosocial outcomes (i.e., more positive self-esteem, lower levels of behavioral problems, more adaptive and less maladaptive cognitive emotion regulation strategies). We also expected ruminative exploration and reconsideration of commitments to be negatively associated with the same set of phenomena. With regard to the person-centered approach, we expected that diffused adolescents would show the least, while foreclosed and achieved adolescents the most favorable psychosocial outcomes.

Fourth, given the similarity of the two models behind DIDS (1) and U-MICS (33), we expected to find significant associations between the corresponding dimensions of the Hungarian versions of the scales and also between the classifications based on H-DIDS and H-U-MICS in both measured identity domains.

## Methods

### Participants and Procedure

The study was approved by the United Ethical Review Committee for Research in Psychology (EPKEB; Reference No.: 2019-82). After receiving their parents' informed consent, all participants filled in the questionnaires in paper-pencil format in classroom settings supervised by undergraduate psychology students serving as research assistants. All data were collected from secondary schools in the South-Western part of Hungary, therefore, data are not representative of Hungarian adolescents in general. Data were collected in several waves and were collapsed to gain the largest possible statistical power. Thus, sample sizes differ for different parts of the Results section. Samples are not independent but overlapping samples.

The sample for testing the factor structure of the Hungarian version of DIDS consisted of 808 adolescents (357 boys and 451 girls). The age of participants was 16.86 years on average (minimum = 14; maximum = 21; SD = 1.35; Skewness = 0.014; SE skewness = 0.086; Kurtosis = −0.688; SE kurtosis = 0.172). The sample for testing the factor structure of the Hungarian version of UMICS consisted of 803 adolescents (353 boys and 450 girls). The age of participants was 16.88 years on average (minimum = 14; maximum = 21; SD = 1.34; Skewness = 0.010; SE skewness = 0.086; Kurtosis = −0.674; SE kurtosis = 0.172).

The sample for testing the validity of the Hungarian version of DIDS 233 adolescents (62 boys and 169 girls; two participants didn't report their gender). The age of the participants was 16.78 years on average (minimum = 14; maximum = 20; SD = 1.60; Skewness = 0.089; SE skewness = 0.160; Kurtosis = −1.161; SE kurtosis = 0.319). The sample for testing the validity of the Hungarian version of UMICS 223 adolescents (56 boys and 165 girls; two participants didn't report their gender). The age of the participants was 16.85 years on average (minimum = 14; maximum = 20; SD = 1.57; Skewness = 0.051; SE skewness = 0.164; Kurtosis = −1.155; SE kurtosis = 0.326).

### Measures

The **Utrecht-Management of Identity Commitments Scale** (U-MICS) (2) was used in the assessment regarding identity processes in the domain of education and friendship. Scales for each identity domains composed of 13 items (commitment: five items, in depth exploration: five items, reconsideration of commitments: three items) rated on a five-point Likert-scale ranging from 1 (completely untrue) to 5 (completely true). Translation in Hungarian was done by the first author. Each translated item was then discussed among the co-authors to develop the final items. Back translation was accomplished by an independent translator, which procedure provided English versions identical in content with the original items of the UMICS.

The **Dimensions of Identity Development Scale** (DIDS) (1) assesses the five identity processes (CM, commitment making; IC, identification with commitment; EB, exploration in breadth; ED, exploration in depth; RE, ruminative exploration) with 25 items. Scales for each identity dimensions composed of five items rated on a five-point Likert-scale ranging from 1 (completely disagree) to 5 (completely agree). Translation in Hungarian was done by the last author. Each translated item was then discussed among the co-authors to develop the final version. Back translation was accomplished by an independent translator, which procedure provided English versions identical in content with the original items of DIDS.

The **Rosenberg Self-esteem Scale** (RSES-H) (36) was used to assess global self-esteem of the participants. The questionnaire/measurement was translated into Hungarian by Sallay et al. (37). The questionnaire consists of 10 items rated on a 4-point scale. The scale proved to be reliable (Cronbach's α = 0.883).

The **Child Behavior Checklist—Youth Self Report (**CBCL-YSR) (38) was assessed to measure behavioral and emotional problems for the previous 6 months. The Hungarian short version of CBCL youth self-report (39, 40) form consists of 44 items. *Social problems* (e.g., “I would rather be alone than with others”), *anxious/depressed* (e.g., “I am afraid I might think or do something bad”), *somatic complaints* (e.g., “I feel overtired without good reason”), *attention problem*s (e.g., “I have trouble concentrating or paying attention”), *aggression* (e.g., “I argue a lot”), and *deviant behavior* (e.g., “I hang around with kids who get in trouble”). Each item is rated on a 0–2 scale (0 “not true,” 1 “somewhat or sometimes true,” and 2 “very true or often true”). Internal reliability was good to excellent for all scales (Cronbach's αs > 0.703), except for Deviant behavior that demonstrated poor reliability (Cronbach's α = 0.493).

The **Cognitive Emotion Regulation Questionnaire** (CERQ) (41) was assessed to evaluate conscious attentional and thinking processes that people use to regulate emotions. The Hungarian version was adapted by Miklósi et al. (42). The questionnaire consists of 36 item measuring nine subscales. The adaptive strategies are *acceptance* (having thoughts of accepting and resigning with regard to what one has experienced)*, positive refocusing* (thinking about positive, happy and pleasant issues instead of thinking about threatening and stressful events), *refocus on planning* (thinking about what steps to do and how to handle the negative event), *positive reappraisal* (having thoughts of giving a positive meaning to the negative events in terms of personal growth), and *putting into perspective* (having thoughts that relativize the seriousness of the negative event comparing it to other events). The less adaptive strategies are *self-blame* (having thoughts of putting the blame on oneself for what one have experienced), *rumination* (having thoughts about the feelings and thoughts associated with the negative events), *catastrophizing* (having thoughts of explicitly emphasizing the negativity of the experience) and *blaming others* (having thoughts of putting the blame on others for what one have experienced). The items are rated on a 5-point Likert scale ranging from 1 (almost never) to 5 (almost always). The scales demonstrated good internal reliability (Cronbach's αs > 0.732), except for Acceptance that had questionable reliability (α = 0.602).

### Statistical Analytical Plan

For statistical analyses, we used IBM SPSS Statistics version 22 and IBM SPSS AMOS version 24. To describe the dimensions of H-DIDS and H-U-MICS, means (Ms) and standard deviations (SDs) were computed. To establish the internal reliability of all measured variables, Cronbach's α values were computed. Crobach's α values above 0.70 were interpreted as indicating acceptable reliability, values between 0.60 and 0.70 as indicating questionable reliability, and values below 0.60 as indicating poor reliability (43). To test the factor structure of the adapted scales, we used confirmatory factor analyses (CFAs). Fit indices were interpreted in accordance with the suggestions of Hu and Bentler (44): a cut-off value close to 0.95 in the case of the Comparative Fit Index (CFI) and the Tucker-Lewis Index (TLI), and a cut-off value close to 0.06 for the Root Mean Square Error of Estimation (RMSEA) result in lower Type II errors without significant increase in Type I errors. Thus, these values can be considered as indices of excellent fit. As a direct comparison of the models, we used Akaike Information Criterion (AIC) values (45), where relatively lower values indicate better fit. To test linear associations between measured variables, we used Pearson's correlations. Besides taking statistical significance at the level of 0.05 into account, only correlation coefficients of |0.20| or higher were interpreted as meaningful.

To classify participants, we followed a two-step procedure previously applied in studies of Finnish (13), Greek [Mastrotheodoros and Motti-Stefandi, (20)], and Italian (46) adaptations of DIDS. In step one, we investigated the visual outputs (dendograms) of hierarchical cluster analyses to determine the number of clusters. Final cluster memberships were determined with k-means cluster analyses performed on the *z*-scores of the variables. To compare the different cluster groups on the measured variables, we used one-way analyses of variance (ANOVAs) with Tukey's honestly significant difference (HSD) *post-hoc* tests. This *post-hoc* tests establish homogenous subsets of groups whose scores are not significantly different from each other (47). Finally, to compare the distribution of participants across clusters based on different sets of variables, we used χ^2^-tests.

## Results

### Testing the Factor Structure of H-DIDS

We used CFAs to test the fit of the six models described in section Methods. According to the results (Table 1), the five-factor model showed the best fit among the tested model, as indicated by the AIC values. This model showed an adequate fit to data, and this fit could be further improved with the implementation of covariances between six pairs of error terms. This final model with error covariances fitted significantly better than the six-factor model (Δχ^2^ = 252.439; Δdf = 1; *p* < 0.001). For the five-factor model with error covariances, factor loadings of the items and correlations between the error terms are shown in Supplementary Figure 1. Correlations between latent variables are shown in Supplementary Table 1.

**Table 1:** Candidate models of the structure of H-DIDS; results of CFAs.

**Models**	**χ^2^**	**df**	**χ^2^/df**	**TLI**	**CFI**	**RMSEA (90% CI)**	**AIC**
Four-factor model: CM and IC in a single factor	1,808.484	269	6.723	0.822	0.841	0.084 (0.081–0.088)	1,970.484
Four-factor model: EB and ED in a single factor	1,640.007	269	6.097	0.842	0.858	0.079 (0.076–0.083)	1,802.007
Four-factor model: EB and RE in a single factor	2,105.829	269	7.828	0.788	0.810	0.092 (0.088–0.096)	2,267.829
Four-factor model: ED and RE in a single factor	1,795.781	269	6.676	0.824	0.842	0.084 (0.080–0.088)	1,957.781
Five-factor model	956.302	260	3.678	0.917	0.928	0.058 (0.054–0.062)	1,136.302
Six-factor model	1,167.106	260	4.489	0.892	0.906	0.066 (0.062–0.070)	1,347.106
Five-factor model with six error covariances	914.667	259	3.532	0.921	0.932	0.056 (0.052–0.060)	1,096.667

*CM, commitment making; IC, identification with commitment; EB, exploration in breadth; ED, exploration in depth; RE, ruminative exploration*.

Means, standard deviations, and internal reliability indices for the five dimensions of H-DIDS are shown in Supplementary Table 1. Except for Exploration in depth, all dimensions showed good to excellent internal reliability. The internal reliability of Exploration in depth proved to be questionable.

The intercorrelations of the five dimensions of H-DIDS were tested with Pearson's correlations (Supplementary Table 1). Dimensions referring to commitment (CM and IC) and dimensions referring to exploration (EB and ED) showed positive correlations with each other with moderate strength, respectively. Ruminative exploration showed significant associations to all the other four dimensions with meaningful strength. It was positively and weakly related to exploration dimensions (EB and ED), whereas it was negatively related to commitment dimensions (CM and IC) with a moderate strength.

### Testing the Factor Structure of H-UMICS

We used CFAs to test the fit of the three models described in section Methods with respect to both educational and relational identity versions of H-UMICS. With regard to both versions, none of the three models showed acceptable fit (Tables 2, 3). On further investigation of factor loadings and error covariances, the possibility of a four-factor model emerged, where the 5-item In depth Exploration factor would be split into two factors: one referring to reflective exploration (i.e., done individually with reflecting upon possibilities; items 6, 7, and 8) and the other referring to socially scaffolded exploration (i.e., discussing possibilities with significant others; items 9 and 10). This model showed acceptable fit that was relatively superior to all three other models (Tables 2, 3). However, the factor referring to socially scaffolded exploration showed questionable to poor internal reliability (Cronbach αs = 0.65 and 0.61 for educational and relational identity, respectively).

**Table 2:** Candidate models of the structure of H-UMICS (educational identity); results of CFAs.

**Models**	**χ^2^**	**df**	**χ^2^/df**	**TLI**	**CFI**	**RMSEA (90% CI)**	**AIC**
One-factor model	1,696.171	65	26.095	0.628	0.690	0.177 (0.170–0.184)	1,774.171
Two-factor model	1,318.074	64	20.595	0.710	0.762	0.156 (0.149–0.164)	1,398.074
Three-factor model	460.966	62	7.435	0.905	0.924	0.090 (0.082–0.097)	544.966
Four-factor model	316.072	59	5.357	0.935	0.951	0.074 (0.066–0.082)	406.072
Three-factor model with seven error covariances	15.658	55	2.849	0.973	0.981	0.048 (0.039–0.057)	254.685

**Table 3:** Candidate models of the structure of H-UMICS (relational identity); results of CFAs.

**Models**	**χ^2^**	**df**	**χ^2^/df**	**TLI**	**CFI**	**RMSEA (90% CI)**	**AIC**
One-factor model	2,056.104	65	31.632	0.599	0.666	0.195 (0.188–0.203)	2,134.104
Two-factor model	1,555.368	64	24.303	0.695	0.750	0.170 (0.163–0.178)	1,635.368
Three-factor model	454.993	62	7.339	0.917	0.934	0.089 (0.081–0.097)	538.993
Four-factor model	388.256	59	5.733	0.938	0.953	0.077 (0.069–0.085)	428.256
Three-factor model with six error covariances	170.640	56	3.047	0.973	0.981	0.051 (0.042–0.059)	266.640

With the implementation of error covariances, the three-factor models could be improved both for educational and relational identity versions. These models with error covariances showed adequate fit, even superior to the four-factor models (Tables 2, 3). Based on these results, we decided to retain the three-factor models for further analyses. For these models, factor loadings of the items and correlations between the error terms and latent factors are shown in Supplementary Figures 2, 3 for the educational identity and relational identity versions, respectively.

Means, standard deviations, and internal reliability indices for the three dimensions of H-UMICS (educational identity) and the three dimensions of H-UMICS (relational identity) are shown in Supplementary Table 2. All dimensions showed good to excellent internal reliability. The intercorrelations of the altogether six dimensions of H-UMICS were tested with Pearson's correlations (Supplementary Table 2). Correlations showed the same pattern for both versions. Commitment was related to both In depth Exploration (positively) and Reconsideration of Commitment (negatively) with moderate strength, while In depth Exploration and Reconsideration of Commitment were unrelated to each other. Across versions, corresponding dimensions showed weak but significant positive correlations in the case of Commitment and In depth Exploration. For Reconsideration, the strength of correlation between educational identity and relational identity was significant but negligible in strength.

### Validation of H-DIDS: Variable-Level and Person-Centered Approaches

At the level of variables, we tested the relationship between the dimensions of H-DIDS and measured variables with Pearson's correlations. Results are shown in Table 4. More positive self-esteem was associated with more intense commitment—both at the level of commitment making and the level of identification with commitment. At the same time, more positive self-esteem was associated with less ruminative exploration. Self-esteem was unrelated to processes of exploration.

**Table 4:** Relationship between the five dimensions of H-DIDS and measured variables; results of Pearson's correlations.

		**CM**	**EB**	**RE**	**IC**	**ED**
RSES		0.479^***^	0.039	−0.461^***^	0.508^***^	−0.057
CBCL	Social problems	−0.241^***^	0.042	0.232^***^	−0.309^***^	0.044
	Anxious	−0.408^***^	0.022	0.384^***^	−0.395^***^	0.124
	Somatic complaints	−0.259^***^	−0.037	0.150^*^	−0.277^***^	−0.015
	Attention problems	−0.278^***^	−0.060	0.312^***^	−0.349^***^	−0.020
	Deviant behavior	−0.148^*^	−0.054	0.157^*^	−0.199^**^	−0.072
	Aggression	−0.121	−0.082	0.151^*^	−0.174^**^	−0.033
CERQ	Self-blame	−0.260^***^	−0.015	0.303^***^	−0.238^***^	0.215^**^
	Acceptance	−0.026	0.107	0.036	−0.014	0.172^**^
	Rumination	−0.110	0.146^*^	0.238^***^	−0.180^**^	0.312^***^
	Positive refocusing	0.224^**^	0.134^*^	−0.107	0.308^***^	0.086
	Refocusing on planning	0.140^*^	0.074	−0.044	0.173^**^	0.133^*^
	Positive reappraisal	0.221^***^	0.107	−0.156^*^	0.259^***^	0.110
	Putting into perspective	0.026	0.113	0.066	0.001	0.152^*^
	Catastrophizing	−0.151^*^	0.041	0.176^**^	−0.062	0.146^*^
	Other-blame	0.017	−0.015	0.052	0.016	0.012

* *p < 0.05*;

** *p < 0.01*;

*** *p < 0.001. CM, commitment making; EB, exploration in breadth; RE, ruminative exploration; IC, identification with commitment; ED, exploration in depth; RSES, Rosenberg self-esteem scale; CBCL, child behavior checklist; CERQ, cognitive emotion regulation questionnaire*.

Regarding problem behaviors, aggression, and deviant behavior were found to be unrelated to the dimensions of identity development. Social problems, anxious symptoms, somatic complaints, and attention problems showed similar associations with the identity development processes. All problems had negative associations with both processes of commitment, while all—except for somatic symptoms—had positive associations with ruminative exploration. The strongest correlations were found for anxious symptoms; these correlations were moderate in strength.

With regard to cognitive emotion regulation strategies, self-blame, rumination, positive refocusing, and positive reappraisal showed significant and weak but meaningful associations with any of the identity development processes. Adaptive strategies (i.e., positive refocusing and positive reappraisal) were associated with more pronounced commitment—both at the level of making commitments and at the level of identifying with them. Negative strategies (i.e., self-blame and rumination) showed somewhat distinct patterns. More self-blame—i.e., blaming yourself for the negative event experienced—was associated with weaker commitments and more intense ruminative and in depth exploration. More ruminative coping strategies—i.e., thinking more about thoughts and feelings related to negative events—were associated with more intensive ruminative and in depth exploration.

**Figure 1:**
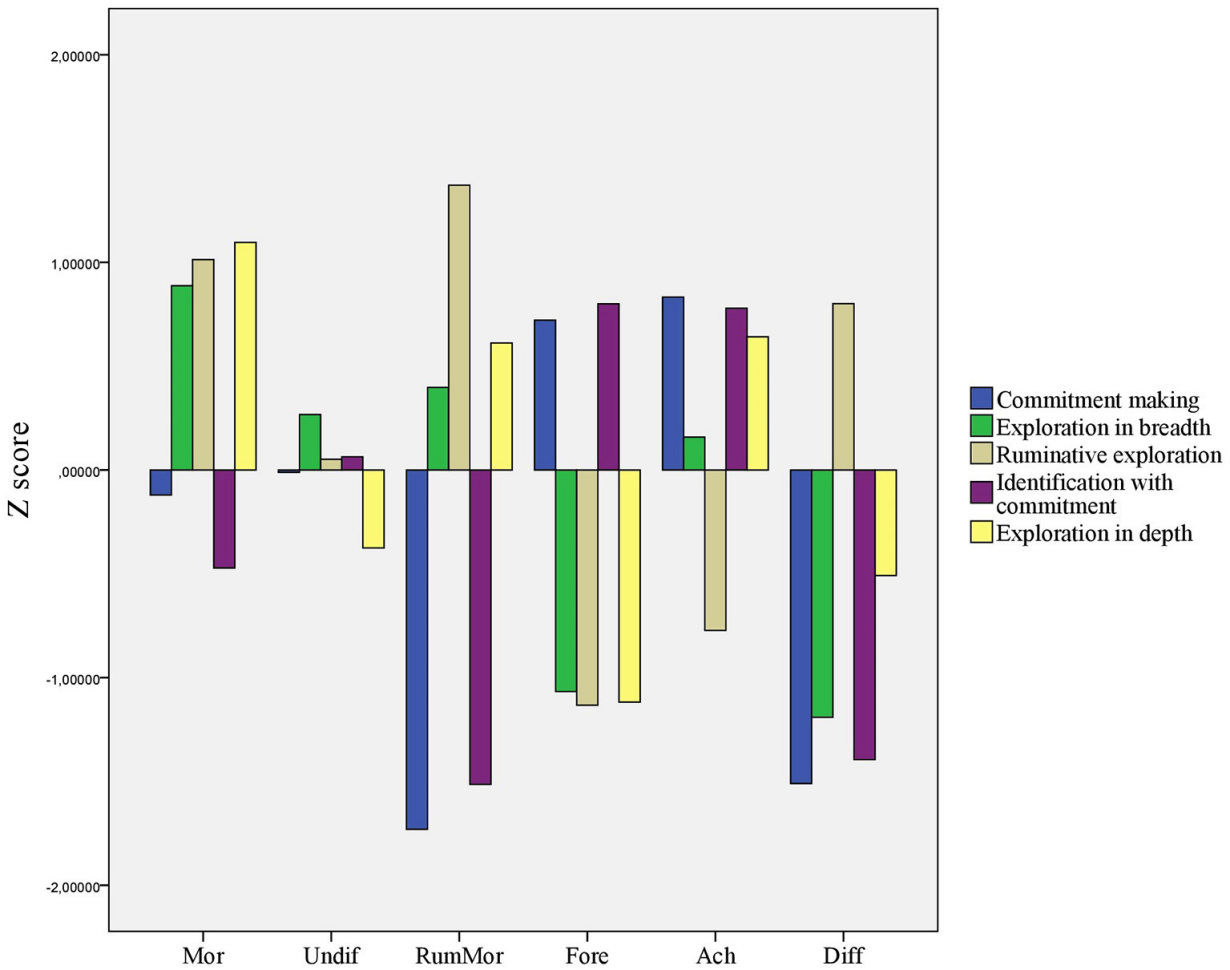
Six clusters based on the *Z-*scores of the five dimensions of H-DIDS; results of k-means clustering. Mor, Moratorium cluster; Undif, Undifferentiated cluster; RumMor, Ruminative Moratorium cluster; Fore, Foreclosure cluster; Ach, Achievement cluster; Diff, Diffusion cluster.

To implement a person centered approach, we used hierarchical cluster analysis to determine the number of clusters. Upon the visual investigation of the dendrogram and results of the original study of Luyckx et al. (1), we decided to have six clusters. Cluster memberships for the six clusters were computed by k-means cluster analysis. *Z*-scores of the dimensions of H-DIDS for the six clusters are shown in Figure 1. Scores with at least one standard deviation away from means were referred to as below or above average scores. Scores with at least a half standard deviation away from means but not further than one standard deviation were referred to as elevated or depressed scores. For labeling the clusters we relied on the works of Luyckx et al. (1) and Marcia (8) whenever it was possible.

Individuals in the first cluster (*n* = 35) had above average scores on all exploration dimensions. Therefore, this cluster was labeled Moratorium. Scores of individuals (*n* = 72) in the second cluster were close to average on all dimensions, thus, this cluster was labeled Undifferentiated. Individuals (*n* = 19) in the third cluster had above average scores on Ruminative exploration, and somewhat elevated scores on the other two exploration dimensions. They had below average scores on both commitment dimensions. We labeled this cluster Ruminative Moratorium. The fourth cluster included individuals (*n* = 39) with elevated scores on both commitment dimensions and below average scores on all three exploration dimensions. We labeled this cluster Foreclosure. The fifth cluster consisted of individuals (*n* = 48) who had elevated scores on both commitment dimensions and on the Exploration in depth dimension. They had a depressed score on Ruminative exploration at the same time. This cluster was labeled Achievement. Finally, individuals (*n* = 20) in the sixth cluster has elevated scores on the Ruminative exploration dimensions, while scores for all the other dimensions were either depressed or below average. This final cluster was labeled Diffusion.

To compare the six clusters on the measured variables, we performed ANOVAs with Tukey's HSD *post-hoc* tests. Results are shown in Table 5. Significant differences were found between the six clusters in the case of self-esteem, social problems, anxious symptoms, somatic complaints, attention problems, deviant behavior, self-blame, rumination, positive refocusing, and catastrophizing. In all cases, the most favorable outcomes—the most positive self-esteem, the least problem behaviors, the most intensive reliance on positive cognitive emotion regulation strategies, and least intensive reliance on negative cognitive emotion regulation strategies—were connected to the Achievement or Foreclosure clusters. These outcomes were significantly more favorable than the outcomes for the Diffusion or Ruminative Moratorium clusters. Exceptionally, in the case of rumination Achievement and Ruminative Moratorium clusters showed the least favorable outcomes. These two clusters formed a homogenous subset with no significant difference.

**Table 5:** Comparison of the six H-DIDS clusters on the measured variables; results of ANOVAs.

		**Mor** **(*****n*** **=** **35)**		**Undif** **(*****n*** **=** **72)**		**RumMor** **(*****n*** **=** **19)**		**Fore** **(*****n*** **=** **39)**		**Ach** **(*****n*** **=** **48)**		**Diff** **(*****n*** **=** **20)**		***F***	***p***	**Tukey's HSD *post-hoc* test**
		**M**	**SD**	**M**	**SD**	**M**	**SD**	**M**	**SD**	**M**	**SD**	**M**	**SD**			
RSES		26.06	4.96	26.40	4.99	20.32	3.74	30.15	4.82	29.27	5.51	22.45	6.14	14.951	<0.001	RumMor, Diff < Diff, Mor < Mor, Undif, Ach < Ach, Fore
CBCL	Social problems	3.14	2.65	3.04	2.33	5.95	3.46	2.38	3.01	2.69	2.37	4.35	2.46	6.109	<0.001	Fore, Ach, Undif, Mor < Ach, Undif, Mor, Diff < Diff, RumMor
	Anxious	6.09	4.12	5.01	3.77	9.00	4.67	2.95	2.69	4.23	3.53	7.85	5.24	9.269	<0.001	Fore, Ach, Undif < Ach, Undif, Mor < Mor, Diff < Diff, RumMor
	Somatic complaints	2.49	2.27	2.14	2.62	4.63	2.97	1.87	2.19	1.96	2.48	3.15	3.63	3.864	<0.001	Fore, Ach, Undif, Mor, Diff < Diff, RumMor
	Attention problems	5.26	2.49	5.35	2.73	7.16	2.79	4.10	2.55	4.06	2.32	6.50	2.78	6.193	<0.001	Ach, Fore, Mor, Undif < Mor, Undif, Diff, RumMor
	Deviant behavior	2.57	1.46	2.60	1.64	3.63	2.31	2.59	1.96	2.19	1.59	3.25	1.68	2.430	0.036	Ach, Mor, Fore, Undif, Diff < Mor, Fore, Undif, Diff, RumMor
	Aggression	2.94	1.80	3.35	2.50	4.37	3.13	2.97	2.25	2.60	1.82	3.40	3.10	1.775	0.119	NA
CERQ	Self-blame	12.03	3.02	10.71	2.84	12.63	2.95	9.77	3.31	10.46	3.54	12.85	3.96	4.552	0.001	Fore, Ach, Undif, Mor < Ach, Undif, Mor, RumMor < Undif, Mor, RumMor, Diff
	Acceptance	12.69	3.00	12.25	2.63	12.68	2.93	11.31	2.87	12.54	2.71	12.20	3.07	1.246	0.288	NA
	Rumination	14.97	4.08	12.26	2.86	14.00	3.43	10.92	3.47	12.73	3.97	12.05	4.02	5.666	<0.001	Fore, Diff, Undif, Ach < Diff, Undif, Ach, RumMor < Ach, RumMor
	Positive refocusing	11.17	4.98	10.76	3.85	10.05	4.45	10.85	4.15	12.69	4.03	9.50	4.30	2.298	<0.05	Diff, RumMor, Undif, Fore, Mor < RumMor, Undif, Fore, Mor, Ach
	Refocusing on planning	14.46	3.59	13.61	2.97	13.11	4.29	13.92	3.56	14.48	3.05	13.15	3.59	0.997	0.421	NA
	Positive reappraisal	13.80	4.25	12.94	2.99	11.26	4.72	13.26	4.05	13.96	3.40	11.90	3.97	2.156	0.060	NA
	Putting into perspective	13.11	3.94	11.89	3.32	12.16	4.44	11.10	3.39	12.35	3.26	12.05	3.63	1.290	0.269	NA
	Catastrophizing	9.97	3.48	9.18	3.32	10.32	3.67	7.77	2.66	8.71	3.69	8.75	4.00	2.274	<0.05	Fore, Ach, Diff, Undif, Mor < Ach, Diff, Undif, Mor, RumMor
	Other-blame	7.49	1.99	8.07	2.95	7.74	2.49	7.38	2.82	7.73	2.66	7.80	3.00	0.409	0.842	NA

*Mor, Moratorium cluster; Undif, Undifferentiated cluster; RumMor, Ruminative Moratorium cluster; Fore, Foreclosure cluster; Ach, Achievement cluster; Diff, Diffusion cluster; RSES, Rosenberg Self-Esteem Scale; CBCL, Child Behavior Checklist; CERQ, Cognitive Emotion Regulation Questionnaire*.

### Validation of H-UMICS: Variable-Level and Person-Centered Approaches

At the level of variables, we tested the relationship between the dimensions of H-UMICS and measured variables with Pearson's correlations. Results are shown in Table 6. Self-esteem was significantly associated with identity processes in the domain of educational identity. More positive self-esteem was associated with more pronounced commitment and less reconsideration of commitment. Neither in depth exploration in the domain of educational identity, nor any identity processes in the domain of relational identity were associated with self-esteem.

**Table 6:** Relationship between the six dimensions of H-UMICS (three dimensions each for educational and relational identity) and measured variables; results of Pearson's correlations.

		**COM_**Ed**_**	**IDE_**Ed**_**	**RECON_**Ed**_**	**COM_**Rel**_**	**IDE_**Rel**_**	**RECON_**Rel**_**
RSES		0.468^***^	0.115	−0.251^***^	0.195^*^	−0.162^*^	−0.112
CBCL	Social problems	−0.271^***^	−0.133^*^	0.243^**^	−0.304^**^	−0.076	0.170^*^
	Anxious	−0.298^***^	0.038	0.194^**^	−0.248^***^	0.152^*^	0.219^**^
	Somatic complaints	−0.237^***^	−0.012	0.094	−0.078	0.154^*^	−0.034
	Attention problems	−0.301^***^	−0.193^**^	0.204^**^	−0.092	0.022	0.047
	Deviant behavior	−0.301^***^	−0.204^**^	0.210^**^	−0.089	0.022	0.047
	Aggression	−0.113	−0.154^*^	0.074	−0.165^*^	0.058	0.112
CERQ	Self-blame	−0.125	0.127	0.070	−0.063	0.174^**^	0.096
	Acceptance	−0.023	0.014	0.052	0.057	0.123	0.009
	Rumination	−0.084	0.181	0.140	−0.030	0.275^***^	0.023
	Positive refocusing	0.234^***^	0.092	−0.013	0.267^***^	0.097	−0.065
	Refocusing on planning	0.191^**^	0.251^***^	−0.030	0.305^***^	0.196^**^	−0.034
	Positive reappraisal	0.321^***^	0.205^**^	−0.027	0.300^***^	0.183^**^	−0.012
	Putting into perspective	0.056	0.047	0.158^*^	0.197^**^	0.221^**^	−0.028
	Catastrophizing	−0.107	0.176^**^	0.166^*^	−0.045	0.208^**^	0.138^*^
	Other-blame	−0.039	0.046	0.181^**^	−0.165^*^	0.043	0.274^***^

* *p < 0.05*;

** *p < 0.01*;

*** *p < 0.001; COM, commitment; IDE, in depth exploration; RECON, reconsideration of commitment; RSES, Rosenberg self-esteem scale; CBCL, child behavior checklist; CERQ, cognitive emotion regulation questionnaire. Subscripts Ed and Rel stand for educational and relational identity, respectively*.

With regard to problem behaviors, commitment (educational identity) was associated with all problem behaviors but aggression. More commitment to education was associated with lower levels of social problems, anxious symptoms, somatic complaints, attention problems, and deviant behavior. More in depth exploration in the domain of education was related to more deviant behavior. More reconsideration of educational commitment was associated with more social problems, attention problems, and deviant behavior. Commitment to friendships and friends was associated to less social problems and to less anxious symptoms. In depth exploration and reconsideration of commitment in the domain of relational identity were unrelated to any of the measured problem behaviors.

Regarding cognitive emotion regulation strategies, more commitment to education was associated with more intensive positive refocusing and positive reappraisal (i.e., thinking more to joyful events when facing adversities and more effort to create positive meanings to negative events, respectively). More in depth exploration of educational issues was associated with more intensive refocusing on planning and positive reappraisal (i.e., thinking more about what actions to take to solve the negative situation and more effort to create positive meanings to negative events, respectively). Reconsideration of educational commitment was unrelated to cognitive emotion regulation strategies.

Being more committed to friendships and friends was associated with more intensive positive refocusing, refocusing on planning, and positive reappraisal (i.e., thinking more to joyful events when facing adversities, thinking more about what actions to take to solve the negative situation, and more effort to create positive meanings to negative events, respectively). More in depth exploration of friendships was associated with more rumination, more putting into perspective, and more catastrophizing (i.e., thinking more about thoughts and feelings related to negative events, emphasizing the relativity of the negative event more, and putting more explicit emphasis on the terror of what they experienced, respectively). Reconsideration of commitment to friendships and friends was associated only with other-blame. More reconsideration of commitments in the relational domain was associated with more thoughts of putting the responsibility for the negative event on the environment or others.

**Figure 2:**
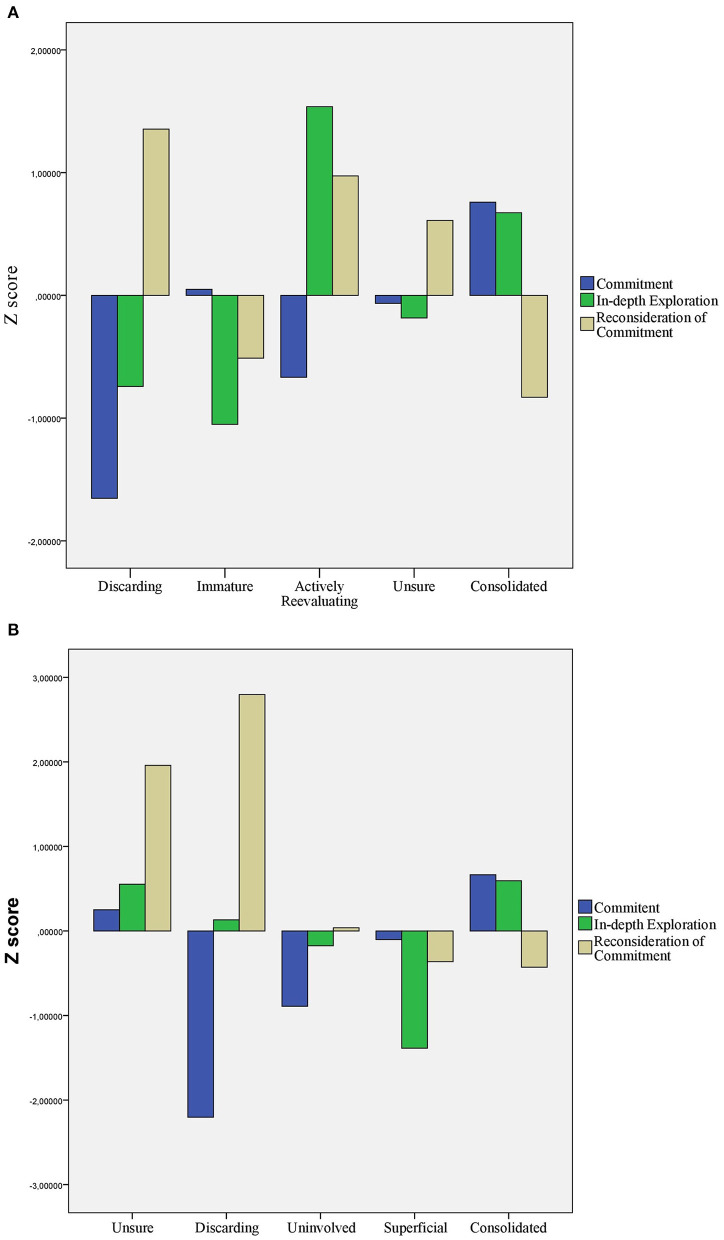
Clusters based on the *Z-*scores of the dimensions of H-UMICS; results of k-means clustering. Clusters in charts **(A,B)** are based on H-UMICS (educational identity) and H-UMICS (relational identity), respectively.

To implement a person centered approach, we used hierarchical cluster analyses—separately for the two identity domains—to determine the number of clusters. Because we were ignorant of any study using clusters based on UMICS, we relied on the visual investigation of the dendrograms. Accordingly, we decided to have five clusters each both for educational and relational identity domains. Cluster memberships for the five-five clusters were computed by k-means cluster analysis. *Z*-scores of the dimensions of H-UMICS (educational identity) for the five clusters and *Z*-scores of the dimensions of H-UMICS (educational identity) for the five clusters are shown in Figures 2A,B, respectively. Scores with at least one standard deviation away from means were referred to as below or above average scores. Scores with at least a half standard deviation away from means but not further than one standard deviation were referred to as elevated or depressed scores. For labeling the clusters we relied on the fact, that the theory behind U-MICS (2, 48, 49) is highly process-oriented. Therefore, labels for the cluster imply processes—despite the fact that we are aware of the cross-sectional nature of our study.

Four clusters showed identical patterns across identity domains. Individuals in the first cluster (*n* = 29 and 7 for educational and relational identity, respectively) had below average scores on Commitment and above average scores on Reconsideration of commitment with either average or depressed scores on In depth exploration. These individuals are reconsidering their commitments with loosening them at the same time. Therefore, we labeled this cluster Discarding (i.e., discarding commitments). Individuals in the second cluster (*n* = 43 and 40 for educational and relational identity, respectively) showed average scores on Commitment, below average scores on In depth Exploration, and slightly depressed scores on Reconsideration of commitment. These individuals are most prominently characterizes by their reluctance to reflect on their choices without being really committed. Although the cluster is similar to foreclosure, focusing on identity development processes, we labeled it Immature for the educational identity domain and Superficial for the relational identity domain. Individuals in the third cluster (*n* = 56 and 15 for educational and relational identity, respectively) had elevated or above average scores on Reconsideration of commitment, but in contrast with individuals in the Discarding cluster, they had average scores on Commitment. These adolescents made thoughts about changing their commitments but without loosening them. Thus, we labeled this cluster Unsure. Individuals in the fourth cluster (*n* = 48 and 108 for educational and relational identity, respectively) had elevated scores on Commitment and In depth exploration and depressed scores on Reconsideration of commitment. These individuals were committed to education and friends while being reflective on these topics at the same time. Therefore, we labeled this cluster Consolidated.

The fifth cluster for the education identity domain included individuals (*n* = 15) who had depressed scores on Commitment, above average scores on In depth exploration, and elevated scores on Reconsideration of commitment. These individuals were actively exploring current commitments without being really committed to them. Additionally, they also made thoughts about discarding these commitments. With emphasizing processes, this cluster was labeled Actively Reevaluating despite its resemblance of moratorium. Individuals in the fifth cluster for the relational identity domain (*n* = 53) had depressed scores on Commitment and average scores on In depth exploration and Reconsideration of commitment. These individuals were uncommitted to friendships and friends without actively reflecting upon or reconsidering the issue. Therefore, this cluster was labeled Uninvolved.

We compared the clusters on the measured variables separately for the two identity domains. For this purpose, we performed ANOVAs with Tukey's HSD *post-hoc* tests. Results for the educational identity domain are shown in Table 7. With regard to self-esteem, significant differences were detected. The *post-hoc* test revealed that adolescents in the Discarding cluster had significantly more negative self-esteem than adolescents from any other cluster. Regarding problem behavior, significant differences were detected between clusters for all kinds of problem behaviors but aggression. For each kind of problem behavior, adolescents from the Consolidated cluster reported the least problems, whereas adolescents from either the Actively Reevaluating or the Discarding clusters reported the most problems. Regarding cognitive emotion regulation strategies, ANOVAs showed significant differences between the clusters for rumination, positive reappraisal, and catastrophizing. *Post-hoc* tests showed that adolescents from the Actively Reevaluating cluster thought significantly more frequently about thoughts and feelings related to negative events than adolescents from any other clusters (rumination). Further, adolescents from the Consolidated cluster made significantly more effort to create positive meanings to negative events than their peers form the Discarding cluster (positive reappraisal). Finally, participants from the Actively Reevaluating cluster put significantly more explicit emphasis on the terror of what they experienced (catastrophizing). Further significant differences between the clusters were indicated by ANOVAs for positive refocusing and refocusing on planning. However, *post-hoc* Tukey's HSD showed only one homogeneous subset of clusters for these variables.

**Table 7:** Comparison of the five H-UMICS (educational identity) clusters on the measured variables; results of ANOVAs.

		**Disc** **(*****n*** **=** **29)**		**Immat** **(*****n*** **=** **43)**		**ActReev** **(*****n*** **=** **15)**		**Unsure** **(*****n*** **=** **56)**		**Cons** **(*****n*** **=** **48)**		***F***	***p***	**Tukey HSD *post-hoc* test**
		**M**	**SD**	**M**	**SD**	**M**	**SD**	**M**	**SD**	**M**	**SD**			
RSES		22.28	6.10	27.12	5.99	24.47	4.34	26.29	4.55	29.13	5.67	10.210	<0.001	Disc, ActReev < ActReev, Unsure. Immat < Unsure, Immat, Cons
CBCL	Social problems	4.31	2.42	2.86	2.75	3.47	2.17	3.50	2.90	2.18	2.20	4.788	0.001	Cons, Immat, ActReev, Unsure < Immat, ActReev, Unsure, Disc
	Anxious	6.62	5.00	4.79	3.83	8.27	3.10	5.50	4.22	3.79	3.43	5.977	<0.001	Cons, Immat, Unsure < Immat, Unsure, Dics < Disc, ActReev
	Somatic complaints	2.83	2.58	2.74	3.27	3.93	3.20	2.32	2.59	1.65	2.10	3.283	0.012	Cons, Unsure, Immat, Disc < Unsure, Immat, Disc, ActReev
	Attention problems	6.66	2.68	5.09	2.69	5.20	2.18	5.14	2.84	3.98	2.36	6.141	<0.001	Cons, Immat, Unsure, ActReev < Immat, Unsure, ActReev, Disc
	Deviant behavior	3.76	1.81	2.72	1.78	2.53	1.88	2.46	1.54	2.20	1.58	4.851	0.001	Cons, Unsure, ActReev, Immat < Immat, Disc
	Aggression	3.66	2.81	3.35	2.19	2.93	1.91	2.80	2.32	2.75	2.17	1.187	0.318	NA
CERQ	Self-blame	11.21	3.40	10.44	3.07	12.67	3.66	11.23	3.45	10.51	2.98	1.848	0.121	NA
	Acceptance	12.48	2.98	12.05	2.75	13.13	2.95	12.05	2.44	12.18	2.71	0.598	0.665	NA
	Rumination	12.90	3.65	11.95	4.15	16.07	2.55	12.89	3.26	12.20	3.40	4.408	0.002	Immat, Cons, Unsure, Disc < ActReev
	Positive refocusing	10.03	4.62	9.70	3.86	10.00	5.03	11.02	3.97	12.05	4.06	2.941	0.021	OHS
	Refocusing on planning	12.93	4.03	12.95	3.02	14.27	4.01	13.70	3.12	14.68	2.81	2.877	0.024	OHS
	Positive reappraisal	11.55	4.41	11.56	3.24	13.07	4.76	13.00	3.16	14.30	3.29	5.751	<0.001	Disc, Immat, Unsure, ActReev < Unsure, ActReev, Cons
	Putting into perspective	12.28	4.17	11.49	3.11	11.87	4.88	12.75	3.14	11.86	3.43	0.924	0.451	NA
	Catastrophizing	9.41	3.41	8.09	3.18	11.73	3.59	9.05	3.18	8.41	3.34	4.178	0.003	Immat, Cons, Unsure, Disc < ActReev
	Other-blame	8.17	3.31	6.93	1.91	7.67	2.23	8.21	3.25	7.49	2.50	1.703	0.150	NA

*Disc, discarding cluster; Immat, immature cluster; ActReev, actively reevaluating cluster; Cons, consolidated cluster; RSES, Rosenberg self-esteem scale; CBCL, Child behavior checklist; CERQ, cognitive emotion regulation questionnaire; OHS, one homogenous subset*.

We also compared clusters on the measured variables for the relational identity domain. Results are shown in Table 8. Regarding self-esteem, adolescents in the Discarding cluster had significantly lower self-esteem than their peers from any other cluster. With regard to problem behaviors, clusters differed significantly in social problems, anxious symptoms, and aggression. *Post-hoc* tests revealed that adolescents from the Discarding cluster reported significantly more social problems than their peers from the Consolidated, Superficial, or Unsure clusters. Discarding adolescents also reported significantly more anxious symptoms than their peers from the Superficial or Consolidated clusters. Finally, participants form the Discarding cluster reported significantly more aggression than their peers from any other cluster.

**Table 8:** Comparison of the five H-UMICS (relational identity) clusters on the measured variables; results of ANOVAs.

		**Unsure** **(*****n*** **=** **15)**		**Disc** **(*****n*** **=** **7)**		**Uninv** **(*****n*** **=** **53)**		**Sup** **(*****n*** **=** **40)**		**Cons** **(*****n*** **=** **108)**		***F***	***p***	**Tukey HSD *post-hoc* test**
		**M**	**SD**	**M**	**SD**	**M**	**SD**	**M**	**SD**	**M**	**SD**			
RSES		26.47	6.42	20.00	2.45	26.00	5.98	28.18	5.58	27.21	5.30	3.693	0.006	Disc < Uninv, Unsure, Cons, Sup
CBCL	Social problems	3.27	2.22	5.57	1.62	3.75	2.24	3.13	3.30	2.39	2.42	4.624	0.001	Cons, Sup, Unsure, Uninv < Uninv, Disc
	Anxious	6.67	4.37	8.57	4.04	6.02	4.24	3.88	4.59	4.62	3.56	3.931	0.004	Sup, Cons, Uninv, Unsure < Uninv, Unsure, Disc
	Somatic complaints	2.07	2.05	3.14	2.61	2.51	2.67	1.83	2.55	2.43	2.81	0.645	0.631	NA
	Attention problems	4.67	2.92	7.29	2.29	5.06	2.71	4.13	2.65	5.02	2.64	2.369	0.054	NA
	Deviant behavior	2.40	1.84	4.00	1.00	2.51	1.74	2.35	1.49	2.66	1.78	1,507	0.201	NA
	Aggression	2.67	1.11	5.86	3.44	3.00	2.41	2.43	2.16	3.09	2.21	3.630	0.007	Sup, Unsure, Uninv, Cons < Disc
CERQ	Self-blame	10.87	3.66	13.86	2.48	10.72	3.22	9.98	2.99	11.18	3.23	2.571	0.039	Sup, Uninv, Unsure, Cons < Cons, Disc
	Acceptance	12.60	3.74	12.57	2.37	12.08	2.37	11.95	2.69	12.32	2.74	0.279	0.891	NA
	Rumination	12.40	3.56	15.00	3.06	12.28	3.57	11.13	3.52	23.33	3.53	3.820	0.005	Sup, Uninv, Unsure, Cons < Cons, Disc
	Positive refocusing	12.27	4.35	7.14	2.34	9.64	3.33	10.25	4.42	11.89	4.22	5.016	0.001	Disc, Uninv, Sup < Uninv, Sup, Cons, Unsure
	Refocusing on planning	14.73	3.49	12.43	2.76	12.38	3.04	13.38	2.89	14.70	3.19	5.894	<0.001	OHS
	Positive reappraisal	14.73	3.26	10.29	3.20	11.94	3.06	11.70	3.96	13.94	3.57	6.523	<0.001	Disc, Sup, Uninv < Sup, Uninv, Cons, Unsure
	Putting into perspective	12.67	3.75	11.71	5.53	11.23	3.26	10.60	3.16	12.96	3.35	4.672	0.001	OHS
	Catastrophizing	10.47	4.26	11.57	3.05	8.40	3.19	8.10	3.07	8.98	3.26	2.931	0.022	Sup, Uninv, Cons, Unsure < Cons, Unsure, Disc
	Other-blame	9.40	2.53	10.43	2.88	7.58	2.94	7.23	2.80	7.44	2.46	3.957	0.004	Sup, Cons, Uninv, Unsure < Unsure, Disc

*Disc, discarding cluster; Uninv, uninvolved cluster; Sup, superficial cluster; Cons, consolidated cluster; RSES, Rosenberg self-esteem scale; CBCL, child behavior checklist; CERQ, cognitive emotion regulation questionnaire; OHS, one homogenous subset*.

Regarding cognitive emotion regulation strategies, ANOVAs showed significant differences between the clusters for all strategies, except for acceptance. However, for refocusing on planning and putting into perspective, only one homogenous subset was detected by *post-hoc* tests. Adolescents from the Discarding cluster blamed themselves more for negative events (self-blame) and thought more frequently about thoughts and feelings related to negative events (rumination) than their peers from the Superficial, Uninvolved, or Unsure clusters. Adolescents from the Consolidated and Unsure clusters reported using more positive refocusing (thinking more to joyful events when facing adversities) and positive reappraisal (putting more effort to create positive meanings to negative events) to cope with negative events than their peers from the Discarding cluster. Discarding adolescents put significantly more explicit emphasis on the terror of what they experienced (catastrophizing) than their peers from the Superficial or Uninvolved clusters. Finally, discarding adolescents put the responsibility for negative events significantly more frequently on the environment or significant others (other-blame) than their peers from the Superficial, Consolidated, or Uninvolved clusters.

### Associations Between H-DIDS and H-UMICS: Variable-Level and Person-Centered Approaches

To test the association between H-DIDS and H-UMICS at the level of variables, we used Pearson's correlations. According to the results (Table 9), the following significant correlations were revealed. More commitment in both domains (as measured by H-UMICS) was associated with more commitment making and identification with commitment and with less ruminative exploration. The correlations were weak to moderate for the educational domain and weak for the relational domain. No other associations were found between H-DIDS and H-UMICS (relational domain). For the educational domain, further significant correlations were revealed. More in depth exploration (as measured by H-UMICS) was associated with more commitment making, exploration in depth, and identification with commitment. More reconsideration of commitments (as measured by H-UMICS) was associated with less commitment making and identification with commitment, and with more ruminative exploration. The correlations between the dimensions of the two domain versions of H-UMICS have been already reported in section Testing the Factor Structure of H-UMICS (Supplementary Table 2).

**Table 9:** The relationship between the five dimensions of H-DIDS and the six dimensions of H-UMICS (educational and relational identity); results of Pearson's correlations.

	**COM_Ed_**	**IDE_Ed_**	**RECON_Ed_**	**COM_Rel_**	**IDE_Rel_**	**RECON_Rel_**
CM	0.459^***^	0.257^***^	−0.361^***^	0.263^***^	−0.040	−0.076
EB	0.031	0.086	0.220^**^	0.020	0.102	−0.063
RE	−0.392^***^	−0.095	0.362^***^	−0.279^***^	0.047	0.141^*^
IC	0.508^***^	0.286^***^	−0.337^***^	0.253^***^	−0.011	−0.018
ED	0.150^*^	0.252^***^	0.118	0.028	0.197^**^	0.106

* *p < 0.05*;

** *p < 0.01*;

*** *p < 0.001. CM, commitment making; EB, exploration in breadth; RE, ruminative exploration; IC, identification with commitment; ED, exploration in depth; COM, commitment; IDE, in depth exploration; RECON, reconsideration of commitment. Subscripts Ed and Rel stand for educational and relational identity, respectively*.

To test the associations at the level of clusters (person-centered approach), we used χ^2^-tests. According to results (see Supplementary Tables 3, 4 for crosstabs), H-DIDS clusters showed a significant overlap with the classification of H-UMICS in the educational identity domain [$\chi_{\left(20\right)}^{2}$ = 111.269; *p* < 0.001]. Adolescents from the Undifferentiated cluster of H-DIDS qualified mostly as members of the Unsure cluster of H-UMICS (educational identity). Adolescent both from Foreclosure and Achievement clusters qualified as members of the Consolidated cluster of H-UMICS (educational identity). However, H-DIDS clusters were independent from the classification of H-UMICS in the relational domain [$\chi_{\left(20\right)}^{2}$ = 23.285; *p* = 0.275]. Moreover, clusters based on the two domain versions of H-UMICS also proved to be unrelated [$\chi_{\left(16\right)}^{2}$ = 22.244; *p* = 0.135; see Supplementary Table 5 for the crosstab].

## Discussion

The aim of the present study was to investigate the psychometric properties and validity of the Hungarian version of two identity scales. Both scales are process-oriented scales that enable us to measure different processes related to identity development. The uniqueness of DIDS is to capture ruminative exploration (1), while in U-MICS a cyclic model of identity is expressed with the introduction of reconsideration of commitments (33). Results are discussed in the same structure as they were reported.

### Factor Structure of H-DIDS

With regard to the possible factor structure of H-DIDS, we tested six candidate models found in the literature: four four-factor models, a five-factor model (1), and a six-factor model (13). The result of CFAs proved that the five-factor model showed an excellent fit; the best among the candidate models. According to these results, both commitment factors and all three exploration factors were independent, collapsing any of those factors resulted in a poorer fit. These results are in accordance with the original conceptualization of the theoretical model behind DIDS (1). We found no proof of the distinction between reflective exploration in depth and reconsideration of commitment—as in the case of the Finnish and Greek versions (13, 20). Exploration in depth was unrelated to any of the commitment dimensions. There is no need to assume two negatively correlated components to plausibly explain these results. It could be simply due to the fact that all exploration dimensions refer to the “work” of identity, while commitment dimensions refer to the—either temporary or relatively permanent—outcome of this exploration process (25). Further, both in breadth and in depth exploration dimensions were positively correlated with ruminative exploration. This might be due to the fact that all three dimensions share a form of reflection. In breadth exploration reflects upon possibilities, in depth exploration upon choices, and ruminative exploration upon despair or lack of choices and possibilities. Thus, we hypothesize that it is not the process of constant reflection on mental processes and their consequences [i.e., rumination itself; for a definition see e.g., (50)] are responsible for the detrimental consequences of rumination (see for previous results; and also see the results in the validation section of this study), but despair. This despair can be elicited by the lack of choices and possibilities as indicated by the negative correlations between in breadth and in depth exploration and ruminative exploration. We hypothesize that this despair is what makes ruminative adolescents vulnerable to depression and anxiety (51) rather than the process of compulsive reflection that they might share with their peers with more favorable outcomes. Therefore, we might consider in furthering the models of identity development to relabel ruminative exploration to aimless or despaired exploration.

### Factor Structure of H-U-MICS

In investigating the factor structure of H-U-MICS, we tested the fit of three candidate models based on previous studies (30, 33). Contrary to our expectations, none of the three tested models had adequate fit. Upon investigation of factor loadings and modification indices, a possible solution with a four-factor model emerged. The factor of in depth exploration was split into two: into a factor with three items reflecting individual reflections about identity elements (reflective exploration) and into another factor with two items reflecting in depth explorations *via* communication with others (socially scaffolded exploration). In the light of its psychosocial roots and the synthesizing function of identity (3, 4, 52, 53), it is rational to assume that while making commitments or even reconsidering them is a *per se* individual, intrapsychic process, exploration takes place in the interpersonal sphere. If we consider the process-orientation of the three-factor model of identity formation (2), we can speculate that socially scaffolded identity precedes reflective exploration. First, the social environment (e.g., parents, peers, and society) offer different possibilities, and then the possibilities are further explored alone in a reflective way. However, these speculations remain hypothetical, because—presumably due to containing only two items—the dimension of socially scaffolded identity showed poor internal reliability in both the educational and the relational identity versions. Therefore, the three-factor model was improved with implementing error covariances, which resulted in a model with acceptable fit for both domain versions of H-U-MICS.

Factors and dimensions showed similar associations for the two domains. Commitment was positively related to in depth exploration and negatively associated with reconsideration of commitment. In depth exploration was unrelated to reconsideration of commitments. These results are in accordance with the results of previous studies (11, 19, 30, 33).

### Classification Based on H-DIDS

Based on the dimensions of H-DIDS, we were able to differentiate between six clusters; as expected. Four of them represented ego identity statuses originally described by Marcia (8): achievement, foreclosure, moratorium, and diffusion. We also found an undifferentiated cluster in which adolescents showed average scores on each dimension. In accordance with the original intentions and results of Luyckx et al. (1), ruminative moratorium emerged as the sixth cluster. None of the adaptation studies that used five dimensions of DIDS (35, 46) was successful in attaining such a cluster. This cluster of ruminative exploration was clearly distinguishes both from the moratorium and the diffusion clusters. In the ruminative moratorium cluster, besides the above average score on ruminative moratorium, adolescents showed elevated scores on the dimensions of in breadth exploration and in depth exploration. Contrasted to that diffused adolescents showed below average scores on the two reflective exploration dimensions—especially on the dimension of in breadth exploration. Moratorium and ruminative moratorium clusters differed both on reflective exploration and commitment dimensions, while ruminative exploration was present in both clusters to the same amount. In moratorium, above average scores on ruminative moratorium was accompanied by above average scores on in breadth and in depth exploration, while these were only elevated for adolescents in the ruminative moratorium cluster. Further, scores for the commitment dimensions were average for adolescents in the moratorium cluster, while being two standard deviations below average for their peers in the ruminative moratorium cluster. Thus, indecision and rumination in the case of adolescents in moratorium might be considered as typical or normal ingredients of this identity development stage or ego identity status, where adolescents are on their way of searching for identity (54). In contrast, rumination and indecision become more pronounced in ruminative moratoriums, where these are accompanied by lack of commitment making and identification with commitment. Using attachment terminology (55), moratoriums have a secure base to explore from (although they might be loosening their commitments), ruminative moratoriums clearly lack this secure base. This speculation is in accordance with results from studies showing that moratoriums experience a family functioning similar to achieved adolescents [e.g., (56, 57)].

### Classification Based on H-U-MICS

With regard to classifications based on the two domain versions of H-U-MICS, adolescents could be classified into five clusters for both the educational and the relational identity versions of H-U-MICS. Thus, the number of clusters is identical to that reported by Crocetti et al. (32) and met our expectations. Four out of the five clusters reported by Crocetti et al. (32) corresponded to the four clusters that showed similar patterns across identity domains. Our consolidated cluster corresponded to achievement, immature (educational identity) and superficial (relational identity) to foreclosure, discarding to moratorium, and unsure to searching moratorium. Although we used a process-oriented terminology for labeling our clusters, in three out of four cases the different labels refer to similar phenomena. However, we consider the label by Crocetti et al. (32) for their moratorium cluster misleading. Moratorium is usually characterized by intensive exploration (8) is not clearly present in this cluster. Adolescents in this cluster are rather loosening their commitments while discarding them. Thus, they move toward the identity vacuum of diffusion rather than toward possible identities, as in the case of moratorium. Hence, we labeled this cluster as discarding.

In our study, with regard to either the educational identity or the relational identity version, no clear-cut diffusion cluster emerged with below average scores on each of the three dimensions (32). Moreover, the fifth clusters were dissimilar for the two identity domain versions. With regard to the educational identity domain, adolescents in the fifth cluster were actively reevaluating their commitments. Above average levels of both in depth exploration and reconsideration of commitment indicated that they really put effort into reflecting on their commitments and into deciding whether or not these commitments suited them. In our view, this pattern is much closer to moratorium with its inherent indecisiveness and uncertainty (8, 54) than the pattern labeled as moratorium by Crocetti et al. (32). With regard to the relational identity domain, adolescents in the fifth cluster showed below average scores on commitment and average scores on in depth exploration and reconsideration of commitment. They were labeled as uninvolved because seemingly their lack of commitment to friends neither motivated them to reconsider the situation or to explore how friendships would suit them. This cluster is most similar to clusters usually labeled as carefree diffusion in studies using DIDS (1, 35, 46).

### Validity of H-DIDS

With regard to the variable-level approach, commitment making and identification with commitment were positively, whereas ruminative exploration was negatively associated with self-esteem. These results are in accordance with previous results [56, (58)]. In our cross-sectional study, we can only speculate on the causal relationship between identity processes and self-esteem. Both directions seem to make sense (59). Positive self-esteem can be a buffer to protect against the vicissitudes of identity formation and contributing to firmer commitments (58). Also having commitments (as opposed to being despaired as in the case of ruminative exploration) can help the development of more positive self-esteem.

Associations between dimensions of identity development and behavioral problems and cognitive emotion regulation strategies showed a similar pattern. Both internalizing and externalizing problems showed negative correlations with dimensions of commitment and positive correlation with ruminative exploration. These results are in accordance with the results of previous studies, where strong intercorrelations were found between identity formation processes and behavioral problems (1, 23, 60). Commitment and ruminative exploration also showed associations with adaptive and maladaptive cognitive emotion regulation strategies. More commitment making and identification with commitment were associated with more adaptive and less maladaptive cognitive emotion regulation strategies, while ruminative exploration was related to less adaptive and more maladaptive cognitive emotion regulation strategies. Given the potential mediator role of emotion regulation between identity processes and behavioral problems (61) and the consequent interrelatedness of identity and emotion regulation across diagnostic groups (62), these results are unsurprising. As exception from the above described pattern, in depth exploration was also positively associated with self-blame and rumination. Given the reflective nature of in depth exploration (1), this process of identity formation inherently includes reflecting upon and being aware of previous commitments. Authentic self-awareness (63) includes being aware of ones strengths and weaknesses at the same time. The latter might lead to self-blame or rumination. The lack of notable associations with cognitive emotion regulation strategies and behavioral problems was observed regarding in-breadth exploration. These results are in accordance with the results of previous studies [31, (58)], which indicate that exploration processes are healthy and adaptive in middle-adolescence, but gradually lose their functionality in the late 20s and they are associated with emotional symptoms with increasing age.

With regard to the person-centered approach, achieved and foreclosed adolescents reported the most favorable outcomes (the most positive self-esteem, the least behavioral problems, and the most adaptive and the least maladaptive cognitive emotion regulation strategies) while ruminative moratoriums and diffused adolescents reported the least favorable outcomes. These results correspond to those of previous studies, where identity clusters with high levels of commitment (i.e., foreclosure and achievement) outperformed identity clusters with low levels of commitment and high levels of ruminative exploration (i.e., diffusion, moratorium, and ruminative moratorium) [e.g., (17, 64–66)]. According to our results, moratorium didn't show as detrimental effects on psychosocial adjustment as either diffusion or ruminative moratorium. Therefore, we speculate that high levels of reflective exploration (i.e., in breadth and especially in depth exploration) might buffer against the negative effects of ruminative exploration [for similar interaction effects see (23)]. While without reflective exploration, ruminative exploration means despair and purposelessness, together with in breadth and in depth exploration, it might indicate nothing more than the temporary insecurity of the normative adolescent crises (54). The maladaptive cognitive emotion regulation strategy of rumination as an exception from this pattern can further strengthen the above line of reasoning. Diffused and foreclosed adolescents showed the lowest levels of rumination in face of adverse life situations, while their peers in the achievement and ruminative moratorium clusters ruminated the most. Rumination defined as the process of constant reflection on mental processes and their consequences (50) is shared by achieved adolescents and their peers in ruminative moratorium. But there is also a main distinction: achieved adolescent with commitments, plans, and life goals have something to reflect upon, while ruminative moratoriums seem to have nothing else but the lack of commitments, plans, and life goals to ruminate about.

### Validity of H-U-MICS

At the level of variables, the following results were obtained. Commitment to education and reconsideration of this commitment were significantly related to self-esteem. More commitment to education was associated with more positive self-esteem, while more reconsideration of commitment was associated with more negative self-esteem. The same pattern was found by Crocetti et al. (32) with regard to self-concept clarity, while Crocetti et al. (10) found the same associations between the dimensions of U-MICS and self-esteem for Arab and Jewish adolescents living in Israel. However, in our study, these associations only emerged for educational identity. With regard to lack of associations in the domain of relational identity, we speculate as follows. Commitment to friendships and friends might have the same importance for adolescents as education (67). At the same time, friendship quality might play a moderating role between commitment and self-esteem (68). We hypothesize that being committed to socially acceptable friends might contribute to positive self-esteem, being committed to socially discarded (i.e., rejected, forbidden) friends might contribute to negative self-esteem.

With regard to behavioral problems, commitment to education was negatively associated with most of the behavioral problems measured, while commitment to friendships was negatively associated only with social problems and anxious symptoms. These results are in accordance with previous studies revealing that externalizing problems interfere with educational identity (69). With regard, to friendship, the quality of friendship might again play an important moderating role (70). Previous studies with U-MICS tended to use composite scores of the two domains, but the importance of commitment in relation with behavioral problems in those studies were similar to our results (10, 11, 71).

With regard to emotion regulation, results in general confirmed those of previous studies showing a positive association between commitment, exploration and adaptive emotion regulation [e.g., (72, 73)]. In contrast to our previously reported associations between identity dimensions and psychosocial functioning, dimensions—especially commitment and in depth exploration—from the relational identity domain produced the more frequent significant correlations as compared to dimension from the educational identity domain. This might be due to methodological issues. We used a scale that measures cognitive emotion regulation strategies in face of negative life events (41). Most negative life events in adolescence are relational in nature (74), therefore, it is unsurprising that we obtained stronger association for cognitive emotion regulation strategies with dimensions from the relational identity domain than with dimensions from the educational identity domain.

In a person-centered approach, results supported the correlational results. Adolescents in the consolidated cluster outperformed their peers in the discarding cluster in psychosocial adjustment and adaptive emotion regulation strategies. These results are in line with those from studies that used a person-centered approach (32, 34, 72). As exceptions, rumination and catastrophizing was significantly characteristic for adolescents in the actively reevaluating cluster than in any other clusters. We speculate that the reflectivity of in depth exploration might potentiate the emergence of negative thoughts (a common characteristic of ruminations and catastrophizing) (41) about negative life events.

### The Association Between H-DIDS and H-U-MICS and the Association Between the Two Identity Domain Versions of H-U-MICS

Finally, the potential associations between H-DIDS and H-U-MICS and the potential association between the two identity domain versions of H-U-MICS were tested. We are ignorant of any studies comparing the two instruments with similar theoretical background (1, 2). Further, based on the fact that scores form the two domains of U-MICS are most frequently used aggregated (10, 19, 31, 32), we are also ignorant of any systematic comparisons between the two identity domain versions of U-MICS.

As for the relation of H-DIDS with the two identity domain versions of H-U-MICS, commitment dimensions in both versions of H-U-MICS were positively associated with commitment dimensions of H-DIDS and negatively with the ruminative exploration dimension of H-DIDS. Accordingly, it seems that commitment—the outcome of the identity work (25)—is quite stable across measures and identity domains. At the same time, even for the commitment dimensions of H-U-MICS, associations with the aforementioned dimensions of H-DIDS were stronger for the educational identity domain. For the educational identity domain version of H-UMICS, in depth exploration and reconsideration of commitment were also significantly and meaningfully correlated with most of the dimensions of H-DIDS. These results are not surprising, given the fact that items of DIDS (1) are formulated with regard to life goals, whereas the educational identity domain version of U-MICS (2) was designed to tap the ideological aspect of identity. Comparing classifications across the two measures also supported the above line of reasoning. Adolescents who were classified based on the five dimensions of H-DIDS were distributed in H-U-MICS (relational identity) clusters by chance. With regard to H-U-MICS (educational identity), a significant overlap with H-DIDS in classification was found. Undifferentiated adolescents (based on H-DIDS) were mostly classified as unsure based on H-U-MICS, whereas achieved and foreclosed adolescents (based on H-DIDS) were mostly classified as consolidated. Based on the conceptualization of identity statuses (8), both foreclosed and achieved adolescents have a solid identity, therefore it is unsurprising that adolescents from both clusters of H-DIDS were classified as consolidated based on the three dimensions of H-U-MICS.

With regard to the two identity domain versions of H-U-MICS, their corresponding dimensions showed weak correlations and classification based on the two versions were independent from each other. On the one hand, these results are important form a methodological aspect. Based on these results, we would advise against the aggregated use of the two domains (10, 19, 31, 32). We also assume that especially reconsideration of commitment could have different meanings for different identity domains. In adolescence, it is more the rule than the exceptions that adolescents question their friendships and change their friends (68). Changing education might be more unusual. This might be partly due to societal expectations. While friendships are voluntary dyadic relationships of affection (75), choices in education is highly effected by parents (76). However, it might be a cultural characteristic of Hungary; a country with considerable levels of uncertainty avoidance (i.e., maintaining rigid codes of belief and behavior and being intolerant of unorthodox behavior and ideas) (77). On the other hand, it is developmentally appropriate for adolescents to be in different phases of identity development (78) that could be another reason for the independence of the two identity domain versions of H-U-MICS.

### Limitations and Conclusions

The current study clearly has its own limitations. The most severe limitations were the use of self-report questionnaires and the cross-sectional design of our study. This latter didn't allow us to evaluate the dynamic nature of identity formation and the direction of the tested associations. Further, our sample cannot be considered representative, as the representativeness was limited by convenience sampling. Another methodological limitation to our study is that we examined a high school sample of adolescents that included individuals ranging in age from 14 to 21 years. Although the identical schooling levels provide a common social norm and expectation toward our participants, the sample cannot be considered homogeneous. However, we decided not to investigate age invariance, because forming group might be arbitrary because of the aforementioned reasons. Future studies should address age invariance of the measures by selecting late primary school students (aged 14–15 years) and university freshmen (aged 18–21 years). This would allow for a clear distinction between the age groups. Furthermore, we did not analyze gender invariance either. This should also be addressed by systematic data collection, i.e., providing a balanced distribution of genders across age groups. The use of item response theory and differential item functioning analyses in future research may further contribute to a more accurate understanding of the psychometric properties of H-DIDS and H-UMICS. Last but not least, in examining the factor structure of U-MICS, the possibility of a four factor model emerged. This raises the question whether in depth exploration can be considered as a homogeneous construct.

In conclusion, the findings of the present study indicate that both H-DIDS and H-U-MICS proved to be reliable and valid instruments to assess identity processes and identity statuses in a Hungarian-speaking context. The parallel work with these two instruments with similar theoretical background (1, 2) gave us the opportunity to compare them with each other, which led us to some theoretical and methodological proposals. Comparing H-DIDS and the two identity dimensions of H-U-MICS revealed that commitment seems to be quite stable across measures and identity domains. Likewise the educational identity domain of H-UMICS also corresponded with H-DIDS as both were designed to grab the ideological aspect of identity. However, the friendship identity domain of H-U-MICS proved to be unrelated to the two ideological domains of identity. This result highlights the divergent developmental dynamics of the ideological and interpersonal identity domains. It is unsurprising because to be at different stages in different domains of development at the same time is developmentally appropriate for adolescents (78). Therefore, for further research, we suggest the assessment of multiple identity domains to get a clearer picture of adolescent identity development, its antecedents and consequences.

## Data Availability Statement

The raw data supporting the conclusions of this article will be made available by the authors, without undue reservation.

## Ethics Statement

The studies involving human participants were reviewed and approved by United Ethical Review Committee for Research in Psychology (EPKEB; Reference No.: 2019-82). Written informed consent to participate in this study was provided by the participants' legal guardian/next of kin.

## Author Contributions

AL and AR: conceptualization and writing—original draft preparation. AL, AR, and NA: methodology. AR, AL, NA, EJ, and BP: item translation process. AL, AR, and EJ: formal analysis and investigation. AL and BP: writing—review and editing and supervision. All authors contributed to the article and approved the submitted version.

## Funding

This project was supported by the European Union and co-financed by the European Social Fund (EFOP-3.6.1.-16-2016-00004—Comprehensive Development for Implementing Smart Specialization Strategies at the University of Pécs). AL was funded by the National Research, Development, and Innovation Office (Grant No. NRDI−138040).

## Conflict of Interest

The authors declare that the research was conducted in the absence of any commercial or financial relationships that could be construed as a potential conflict of interest.

## Publisher's Note

All claims expressed in this article are solely those of the authors and do not necessarily represent those of their affiliated organizations, or those of the publisher, the editors and the reviewers. Any product that may be evaluated in this article, or claim that may be made by its manufacturer, is not guaranteed or endorsed by the publisher.

## Supplementary Material

Supplementary Figure 1 The five-factor model of H-DIDS; results of a CFA. All factor loadings and correlations are significant at *p* < 0.05. Correlations between factors are omitted for clarity and are presented in Table 2. CM, Commitment making; EB, Exploration in breadth; RE, Ruminative Exploration; IC, Identification with commitment; ED, Exploration in depth.

Supplementary Figure 2 The three-factor model of H-UMICS (educational identity); results of a CFA. All factor loadings and correlations are significant at *p* < 0.05. COM, Commitment; IDE, In-depth Exploration; RECON, Reconsideration of Commitment.

Supplementary Figure 3 The three-factor model of H-UMICS (relational identity); results of a CFA. All factor loadings and correlations—both between factors and between error terms—are significant at *p* < 0.05. COM, Commitment; IDE, In-depth Exploration; RECON, Reconsideration of Commitment.

Supplementary Table 1 The interrelations of the five dimensions of H-DIDS; descriptives, internal reliability indices and results of Pearson's correlations. Note: **p* < 0.05; ***p* < 0.01; ****p* < 0.001; CM = Commitment making; EB = Exploration in breadth; RE = Ruminative Exploration; IC = Identification with commitment; ED = Exploration in depth. Correlation between the corresponding latent variables of the final CFA with error covariances are in parentheses.

Supplementary Table 2 The interrelations of the six dimensions of H-UMICS (three dimensions each for educational and relational identity); descriptives, internal reliability indices and results of Pearson's correlations. Note: **p* < 0.05; ***p* < 0.01; ****p* < 0.001; COM = Commitment; IDE = In depth Exploration; RECON = Reconsideration of Commitment. Subscripts Ed and Rel stand for educational and relational identity, respectively. The corresponding dimensions of H-UMICS educational and relational identity scales are bolded.

Supplementary Table 3 The relationship between H-DIDS and H-UMICS (educational identity) classifications. Note: Undif = Undifferentiated cluster; Diff = Diffusion cluster; Ach = Achievement cluster; Fore = Foreclosure cluster; Mor = Moratorium cluster; RumMor = Ruminative Moratorium cluster; ActReev = Actively Reevaluating cluster; Immat = Immature cluster; Cons = Consolidated cluster; Disc = Discerning cluster.

Supplementary Table 4 The relationship between H-DIDS and H-UMICS (relational identity) classifications. Note: Undif = Undifferentiated cluster; Diff = Diffusion cluster; Ach = Achievement cluster; Fore = Foreclosure cluster; Mor = Moratorium cluster; RumMor = Ruminative Moratorium cluster; Uninv = Uninvolved cluster; Disc = Discarding cluster; Sup = Superficial cluster; Cons = Consolidated cluster.

Supplementary Table 5 The relationship between H-UMICS (educational identity) and H-UMICS (relational identity) classifications. Note: Uninv = Uninvolved cluster; Disc = Discarding cluster; Sup = Superficial cluster; Cons = Consolidated cluster; ActReev = Actively Reevaluating cluster; Immat = Immature cluster.
