# Supplementary figures and images for: The Assessment of Dual-Cycle Identity Models Among Secondary School Students: The Hungarian Adaptation of DIDS and U-MICS
Source: Front Psychiatry. 2022 Mar 21;13:804529. doi: 10.3389/fpsyt.2022.804529 (PMC8977606; doi:10.3389/fpsyt.2022.804529)

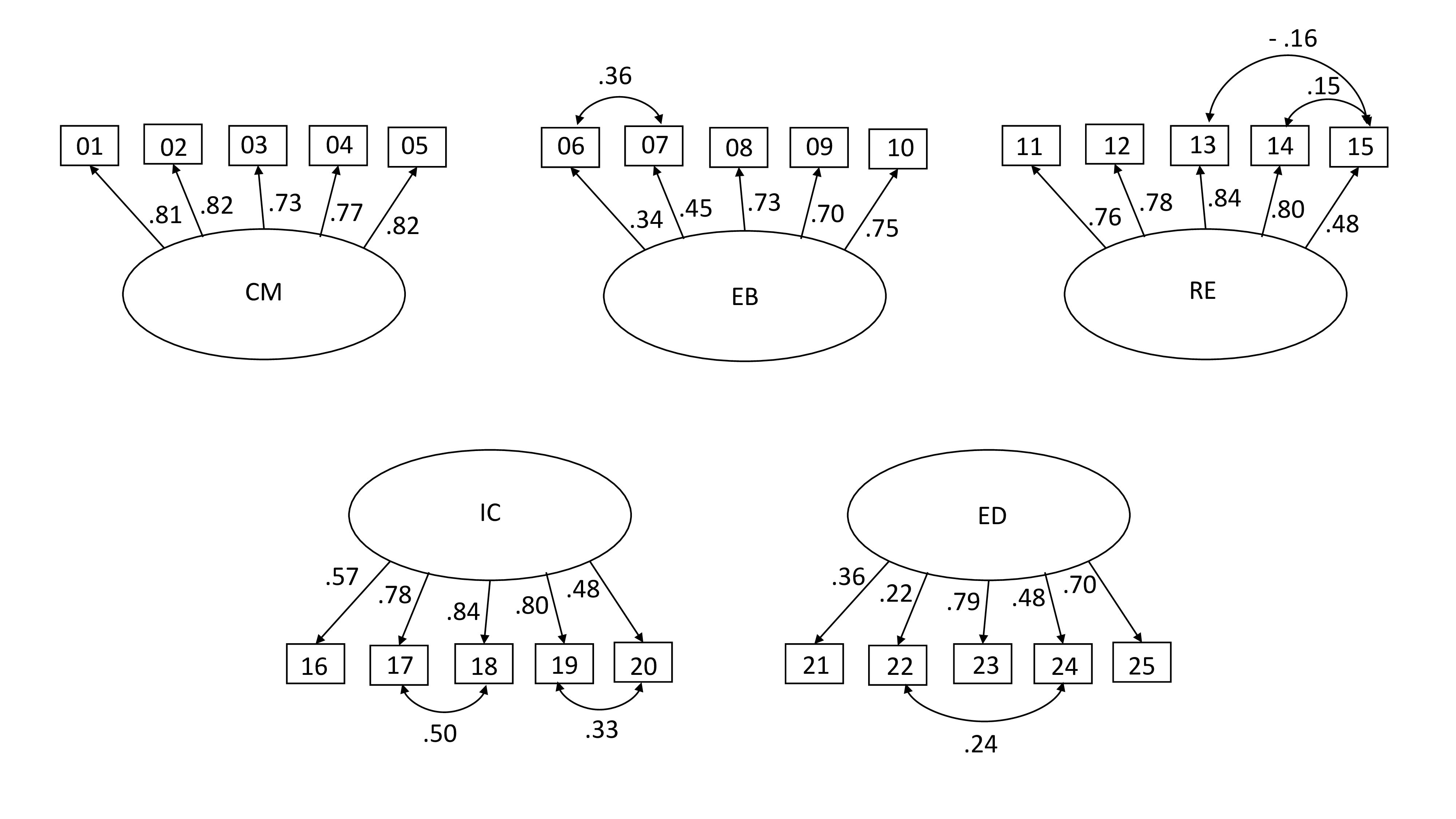

Supplement: Supplementary Figure 1 — The five-factor model of H-DIDS; results of a CFA. All factor loadings and correlations are significant at p < 0.05. Correlations between factors are omitted for clarity and are presented in Table 2. CM, Commitment making; EB, Exploration in breadth; RE, Ruminative Exploration; IC, Identification with commitment; ED, Exploration in depth. [file Image_1.JPEG]

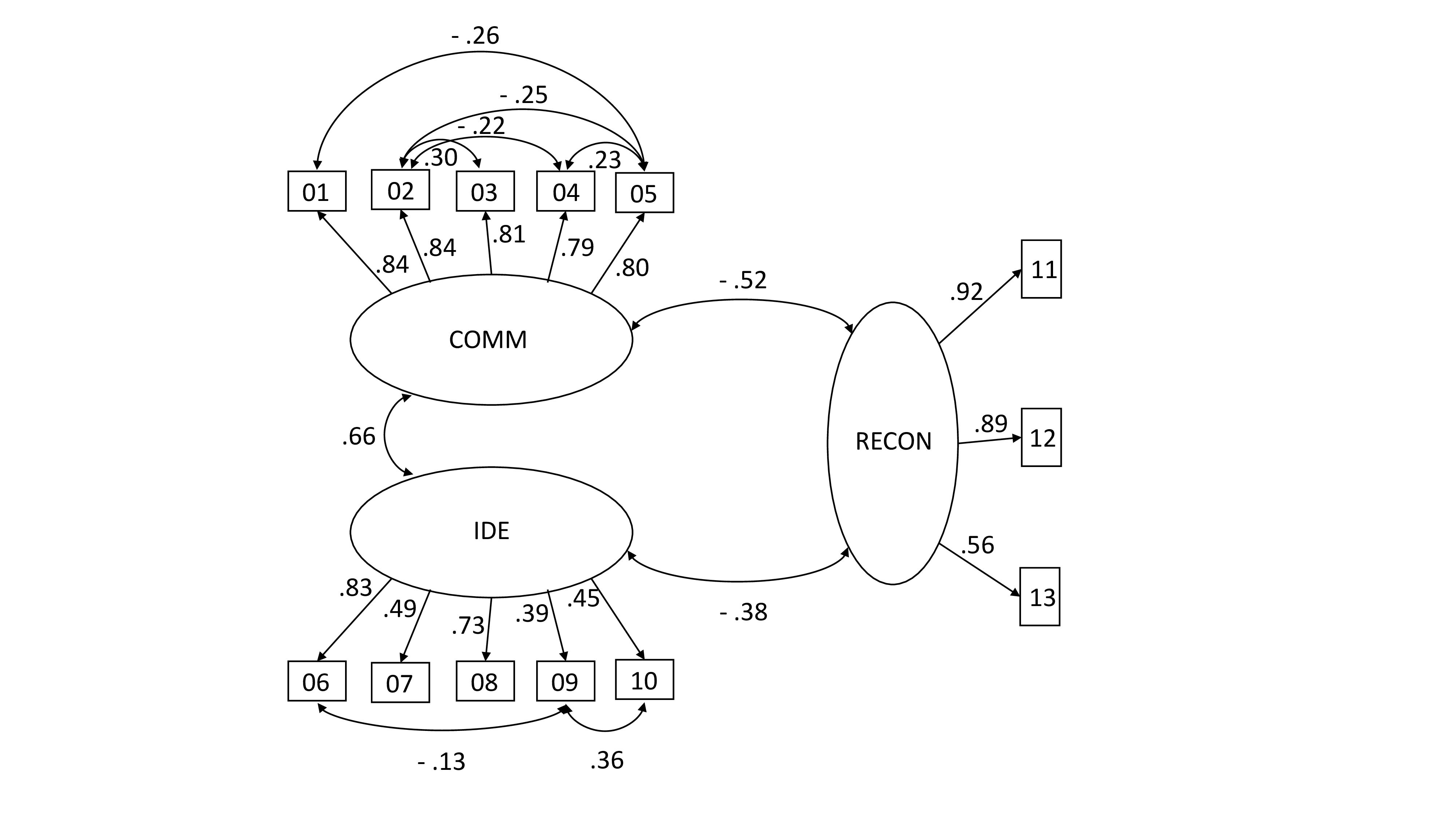

Supplement: Supplementary Figure 2 — The three-factor model of H-UMICS (educational identity); results of a CFA. All factor loadings and correlations are significant at p < 0.05. COM, Commitment; IDE, In-depth Exploration; RECON, Reconsideration of Commitment. [file Image_2.JPEG]

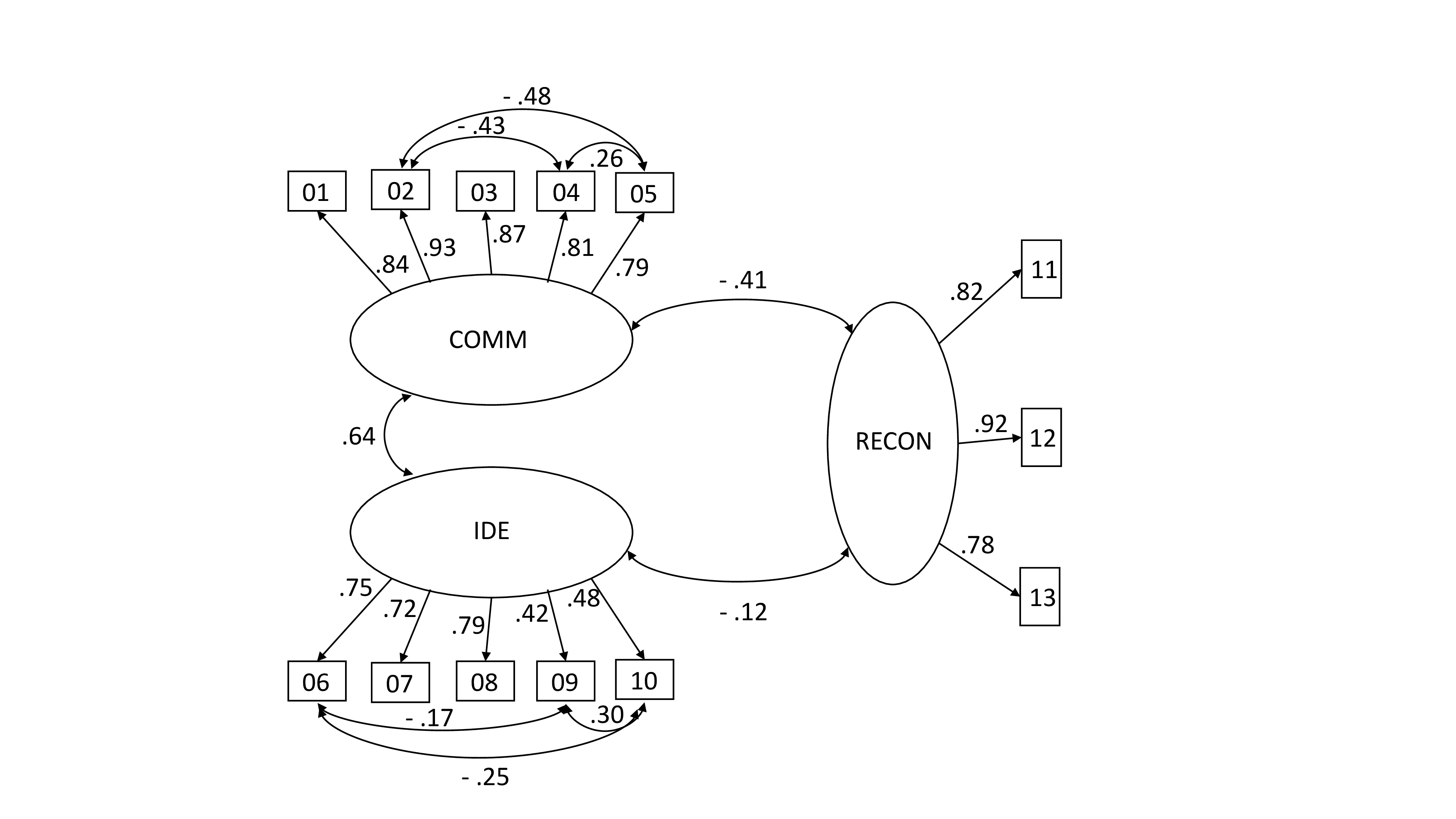

Supplement: Supplementary Figure 3 — The three-factor model of H-UMICS (relational identity); results of a CFA. All factor loadings and correlations—both between factors and between error terms—are significant at p < 0.05. COM, Commitment; IDE, In-depth Exploration; RECON, Reconsideration of Commitment. [file Image_3.JPEG]
