# Supplementary material for: The Assessment of Dual-Cycle Identity Models Among Secondary School Students: The Hungarian Adaptation of DIDS and U-MICS
Source: Front Psychiatry. 2022 Mar 21;13:804529. doi: 10.3389/fpsyt.2022.804529 (PMC8977606; doi:10.3389/fpsyt.2022.804529)
Supplement: Supplementary Table 1 — The interrelations of the five dimensions of H-DIDS; descriptives, internal reliability indices and results of Pearson's correlations. Note: *p < 0.05; **p < 0.01; ***p < 0.001; CM = Commitment making; EB = Exploration in breadth; RE = Ruminative Exploration; IC = Identification with commitment; ED = Exploration in depth. Correlation between the corresponding latent variables of the final CFA with error covariances are in parentheses. [file Table_1.docx]

**Supplementary Material**

Table S1. The interrelations of the five dimensions of H-DIDS; descriptives, internal reliability indices and results of Pearson’s correlations.

|  | CM | EB | RE | IC | ED |
| --- | --- | --- | --- | --- | --- |
| CM |  | .029  (- .059) | - .574***  (- .684***) | .692***  (.813***) | .010  (- .141**) |
| EB |  |  | .315***  (.422***) | - .031  (- .130**) | .419***  (.547***) |
| RE |  |  |  | - .584***  (­- .706***) | .337***  (.537***) |
| IC |  |  |  |  | -.102**  (- .331***) |
| M (SD) | 19.24 (3.63) | 18.58 (3.64) | 12.83 (4.96) | 18.52 (4.15) | 16.23 (3.90) |
| Cronbach’s | .89 | .75 | .85 | .87 | .66 |
| Note: * p < .05; ** p < .01; *** p < .001; CM = Commitment making; EB = Exploration in breadth; RE = Ruminative Exploration; IC = Identification with commitment; ED = Exploration in depth. Correlation between the corresponding latent variables of the final CFA with error covariances are in parentheses. | | | | | |

Table S2. The interrelations of the six dimensions of H-UMICS (three dimensions each for educational and relational identity); descriptives, internal reliability indices and results of Pearson’s correlations.

|  | COM_Ed_ | IDE_Ed_ | RECON_Ed_ | COM_Rel_ | IDE_Rel_ | RECON_Rel_ |
| --- | --- | --- | --- | --- | --- | --- |
| COM_Ed_ |  | .439*** | - .474*** | **.247***** | .138*** | - .031 |
| IDE_Ed_ |  |  | - .192*** | .217*** | **.276***** | .070* |
| RECON_Ed_ |  |  |  | - .027 | .081* | **.137***** |
| COM_Rel_ |  |  |  |  | .518*** | - .378*** |
| IDE_Rel_ |  |  |  |  |  | - .032 |
| M (SD) | 16.41 (4.41) | 15.18 (3.87) | 7.77 (3.34) | 19.20 (4.67) | 17.23 (4.23) | 4.47 (2.59) |
| Cronbach’s α | .91 | .74 | .83 | .92 | .76 | .87 |
| Note: * p < .05; ** p < .01; *** p < .001; COM = Commitment; IDE = In depth Exploration; RECON = Reconsideration of Commitment. Subscripts Ed and Rel stand for educational and relational identity, respectively. The corresponding dimensions of H-UMICS educational and relational identity scales are bolded. | | | | | | |

Table S3. The relationship between H-DIDS and H-UMICS (educational identity) classifications.

|  | | H-UMICS clusters | | | | | Total |
| --- | --- | --- | --- | --- | --- | --- | --- |
|  |  | Unsure | ActReev | Immat | Cons | Disc |  |
| H-DIDS clusters | Undif | 25 | 1 | 14 | 10 | 6 | 56 |
|  | Diff | 5 | 0 | 3 | 3 | 8 | 19 |
|  | Ach | 8 | 2 | 5 | 27 | 0 | 42 |
|  | Fore | 5 | 0 | 8 | 21 | 5 | 39 |
|  | Mor | 10 | 10 | 6 | 6 | 2 | 34 |
|  | RumMor | 5 | 1 | 0 | 2 | 9 | 17 |
| Total | | 58 | 14 | 36 | 69 | 30 | 207 |
| Note: Undif = Undifferentiated cluster; Diff = Diffusion cluster; Ach = Achievement cluster; Fore = Foreclosure cluster; Mor = Moratorium cluster; RumMor = Ruminative Moratorium cluster; ActReev = Actively Reevaluating cluster; Immat = Immature cluster; Cons = Consolidated cluster; Disc = Discerning cluster. | | | | | | | |
|  | | | | | | | |

Table S4. The relationship between H-DIDS and H-UMICS (relational identity) classifications.

|  | | H-UMICS clusters | | | | | Total |
| --- | --- | --- | --- | --- | --- | --- | --- |
|  |  | Uninv | Disc | Unsure | Sup | Cons |  |
| H-DIDS clusters | Undif | 17 | 1 | 4 | 11 | 23 | 56 |
|  | Diff | 5 | 2 | 1 | 5 | 6 | 19 |
|  | Ach | 12 | 0 | 3 | 4 | 23 | 42 |
|  | Fore | 6 | 0 | 1 | 7 | 25 | 39 |
|  | Mor | 7 | 2 | 4 | 5 | 16 | 34 |
|  | RumMor | 4 | 2 | 0 | 3 | 8 | 17 |
| Total | | 51 | 7 | 13 | 35 | 101 | 207 |
| Note: Undif = Undifferentiated cluster; Diff = Diffusion cluster; Ach = Achievement cluster; Fore = Foreclosure cluster; Mor = Moratorium cluster; RumMor = Ruminative Moratorium cluster; Uninv = Uninvolved cluster; Disc = Discarding cluster; Sup = Superficial cluster; Cons = Consolidated cluster. | | | | | | | |

Table S5. The relationship between H-UMICS (educational identity) and H-UMICS (relational identity) classifications.

|  | | H-UMICS (educational identity) clusters | | | | | Total |
| --- | --- | --- | --- | --- | --- | --- | --- |
|  |  | Unsure | ActReev | Immat | Cons | Disc |  |
| H-UMICS (relational identity) clusters | Uninv | 13 | 4 | 11 | 15 | 8 | 51 |
|  | Disc | 2 | 2 | 0 | 0 | 3 | 7 |
|  | Unsure | 6 | 0 | 2 | 5 | 0 | 13 |
|  | Sup | 9 | 0 | 7 | 12 | 7 | 35 |
|  | Cons | 28 | 8 | 16 | 37 | 12 | 101 |
| Total | | 58 | 14 | 36 | 69 | 30 | 207 |
| Note: Uninv = Uninvolved cluster; Disc = Discarding cluster; Sup = Superficial cluster; Cons = Consolidated cluster; ActReev = Actively Reevaluating cluster; Immat = Immature cluster. | | | | | | | |
